# Factors that influence the provision of enteral feeding for critically ill children: a qualitative evidence synthesis

**DOI:** 10.1186/s40795-025-01077-3

**Published:** 2025-05-19

**Authors:** Elisabeth Søiland, Claire Glenton, Susan Munabi-Babigumira, Lena Victoria Nordheim, Suzgika Lakudzala, Idriss Ibrahim Kallon, Celeste Naude, Amanda Brand, Simon Lewin, Nyanyiwe Masingi Mbeye

**Affiliations:** 1 Section for Evidence-Based Practice, Department of Health and Functioning, Western Norway University of Applied Sciences, Bergen, Norway; 2 Evidence Informed Decision-making Centre, Department of Community and Environmental Health, School of Global and Public Health, Kamuzu University of Health Sciences, Blantyre, Malawi; 3 Centre for Evidence-based Health Care, Department of Global Health, Division of Epidemiology and Biostatistics, Faculty of Medicine and Health Sciences, Stellenbosch University, Stellenbosch, South Africa; 4 Department of Health Sciences Ålesund, Faculty of Medicine and Health Sciences, Norwegian University of Science and Technology, Ålesund, Norway; 5 Health Systems Research Unit, South African Medical Research Council, Cape Town, South Africa; 6 Centre for Epidemic Interventions Research (CEIR), Norwegian Institute of Public Health, Oslo, Norway

**Keywords:** Enteral feeding, Critical illness, Children, Caregivers, Healthcare personnel, Qualitative research, Systematic review, Qualitative evidence synthesis, Nutrition

## Abstract

**Background:**

This review aims to explore factors associated with the provision of enteral nutrition in critically ill children in intensive care. Critically ill children are at risk of becoming malnourished, which can lead to poorer outcomes such as longer hospital stays, increased readmissions, and increased mortality. Optimal enteral feeding can help prevent and manage malnutrition.

**Methods:**

We searched MEDLINE, CINAHL, Embase, and Scopus from inception to July 3, 2023. We included qualitative studies and mixed-methods studies. As we anticipated few studies in critical care settings, we included studies that focused on experiences and perceptions of any stakeholders involved in enteral feeding in children in any hospital setting to provide indirect evidence. Titles, abstracts and potentially eligible full-text records were independently assessed. We extracted descriptive data and used a thematic analysis approach to synthesise the study data. We used the GRADE-CERQual approach to assess our confidence in each finding.

**Results:**

We included 14 studies, with four from critical care settings. Where we had concerns that the context of the studies may be less relevant to the setting, intervention and population of interest, we indicated this inour GRADE-CERQual assessment. We had moderate, low or very low confidence in our findings, in part due to the indirectness of the evidence. Moderate confidence findings indicated that parents were concerned about discomfort and side effects, and that a lack of resources could be a barrier to providing optimal nutritional support. Low confidence findings suggested that healthcare workers lacked the knowledge and skills to provide adequate nutritional support, and that receiving support, information, and participating in decision-making around enteral feeding was important to some parents.

**Conclusions:**

Our review points to several factors that may influence the provision of enteral feeding for critically ill children. More qualitative research on healthcare workers’ nutritional support practices, particularly in critical care settings, is needed to provide a better understanding of the barriers to optimal enteral feeding in critically ill children.

**Supplementary Information:**

The online version contains supplementary material available at 10.1186/s40795-025-01077-3.

## Introduction

Malnutrition is common among hospitalised critically ill children [1]. Moderate to severe malnutrition at paediatric intensive care unit (PICU) admission is prevalent worldwide [2], and may be exacerbated by critical illness or injury. In addition, critically ill children are also at risk of nutritional depletion due to the chain reaction of metabolic and hormonal changes produced by critical illness [3] and may become malnourished during their hospital stay due to feeding challenges and suboptimal nutritional support [4–6].

Enteral nutrition is considered the preferred way of providing nutritional support for critically ill children when indicated [7, 8]. Among critically ill children, enteral feeding most commonly involves a nasogastric feeding tube (NGT) inserted through the nose into the stomach as this is the simplest to administer and can be initiated most quickly [9]. Enteral feeds can also be provided by a feeding tube to the duodenum or jejunum [7, 8]. The evidence is uncertain about the effects of nutritional support on outcomes related to mortality and morbidity, or other outcomes such as length of hospital stay, ventilator days or unexpected adverse events [10–12], particularly around the timing of initiation. Nevertheless, preventing undernourishment during illness is essential as malnutrition can worsen outcomes in critically ill children, including length of hospital stay, need for mechanical ventilation, morbidity and mortality [13–15].

When implementing enteral feeding, a focus on nutritional support is likely to compete with other clinical considerations [16]. Tube feeding may be interrupted due to diagnostic tests or procedures, fluid restriction, tube intolerance or tube failure [17, 18]. Among critically ill *adult* patients, providers’ access to training, education and support; access to resources; and perceptions regarding their own responsibilities are factors that can influence how enteral feeding is implemented [19]. An earlier systematic review suggested the implementation of nutritional protocols to improve nutritional support practices could improve the timeliness of feeding initiation, or help providers to reach nutritional goals for feeding [20]. However, the evidence base is likely incomplete as this review was published ten years ago, and the certainty of evidence is unclear.

This qualitative evidence synthesis (QES) aims to explore factors that influence the provision of enteral feeding for critically ill children between the ages of 0 and 18 in paediatric intensive care settings, informed by views, attitudes, experiences and behaviours of the stakeholders involved. The QES was done as part of the Global Evidence, Local Adaptation (GELA) project to inform a national clinical guideline in Malawi. The GELA project aims to produce evidence-informed, locally relevant guideline recommendations for newborn and child health in Malawi, Nigeria and South Africa using best practice methods for guideline adaptation to improve efficiencies in low resource settings. Specifically, guideline development methods for the GELA project were based on GRADE-ADOLOPMENT, an Evidence-to-Decision (EtD) framework-based approach to adopt, adapt or create contextualized recommendations from source guidelines and evidence syntheses [21]. The topic of early versus delayed enteral feeding in critically ill children was identified as requiring a recommendation to guide clinical practice in Malawi [22]. Since a scoping search indicated a paucity of qualitative studies on the specific question of early versus delayed enteral feeding and few studies in paediatric critical care settings, we decided to focus our QES on the provision of enteral feeding for critically ill children regardless of timing, and include studies addressing enteral feeding in any hospital setting. Guideline development took place in Malawi in 2024. The GRADE Evidence-to-Decision (EtD) framework helps decision-makers consider the effectiveness of an intervention, as well as its resource, acceptability, feasibility and equity implications [23]. The findings from this QES were used to inform decisions related to the acceptability, feasibility and equity criteria of the EtD framework and to develop implications for practice.

## Materials & methods

Before beginning the QES, and aligned with the GRADE-ADOLOPMENT approach [21], we searched Epistemonikos (epistemonikos.org) for existing QES, broad syntheses and structured summaries to identify a relevant QES we could use or update for the guideline development process (see the search strategy in Additional file 1). We were, however, unable to identify any QES covering our topic of interest and therefore undertook a new QES.

When preparing this review, we used the Cochrane Qualitative Evidence Syntheses: Protocol and review template v1.4 [24] and prospectively registered it on the international prospective register of systematic reviews, PROSPERO (https://www.crd.york.ac.uk/PROSPERO/view/CRD42023432643), see Additional file 2. Differences between the protocol and QES are described in Additional file 3.

### Review co-production with relevant stakeholders

The topic of this review was determined through a process using best practice priority-setting methods to identify priorities for guidelines in newborn and child health in South Africa, Malawi and Nigeria [22]. These methods included stakeholder consultations, online priority-setting surveys and consensus meetings with representatives from the Ministry of Health of Malawi and with Malawian clinical experts, guided by a national Steering Group.

To further refine the scope of this review, we invited clinicians from hospital settings where enteral feeding is provided to children to participate in a structured discussion using the TRANSFER conversation guide [25]. During this discussion, we asked them to identify contextual factors that they believe are likely to influence the review findings. These were factors tied to the clinical setting, the type of healthcare workers providing care, and the age of the study. We considered these factors when carrying out our analysi*s* and when assessing the ‘relevance’ component of our GRADE-CERQual assessment (Confidence in the Evidence from Reviews of Qualitative research) [26] (see below). We also consulted these stakeholders when developing our ‘Implications for practice’ section.

### Criteria for considering studies for this review

#### Types of studies

We searched for and included primary studies that employed qualitative research designs [27]. Our search focused on studies utilizing qualitative methods for both data collection and analysis. Studies that gathered data using qualitative methods but did not use qualitative methods for the analysis were excluded. Due to time constraints, we only included published studies and studies in English.

We included mixed-methods studies where it was possible to extract the data that were collected and analysed using qualitative methods.

We included studies regardless of whether they were conducted alongside studies of the effectiveness of early enteral nutrition in critically ill children.

We did not exclude studies based on our assessment of methodological limitations. We used this information about methodological limitations to assess our confidence in the review findings.

#### Topic of interest

The aim of this review was to provide evidence for a prioritised question around enteral feeding for critically ill children from birth to 18 years in paediatric intensive care. However, since our scoping search retrieved very few references, we decided to broaden our search in two ways: (1) we also included studies of children receiving enteral feeding in any general paediatric hospital setting, and (2) we included studies from neonatal intensive care (NICU) settings. Despite differences in the clinical situations and practices, we believed some aspects of caregivers’ and healthcare workers’ experiences related to enteral feeding in these settings could be transferred to the PICU setting. Additionally, evidence related to system-level factors (e.g., training or management) or some factors related to workplace practices (such as the use of nutritional tools or protocols) could be relevant across settings. Our aim remained the same, but we included studies from these settings to provide indirect evidence. Where we had concerns that studies from non-intensive care or NICU settings may be less relevant and transferable for each specific finding, we have indicated this in our GRADE-CERQual assessment of relevance [26]. Findings based on indirect evidence have received lower confidence ratings.

By ‘caregivers’, we mean anyone who is directly involved in caring for the child or making the decision of accepting the intervention on behalf of the child. By ‘healthcare workers’ we mean any cadre of healthcare workers involved in the provision of enteral nutrition for children, as well as any other staff involved in supporting the provision of enteral nutrition for children, e.g. administrative, managerial or supervisory staff. By ‘enteral feeding’ we mean the delivery of nutrition directly into the GI tract through a tube, e.g., a nasogastric tube (NGT), or a nasoduodenal or nasojejunal tube.

We excluded studies where the primary focus was on child enteral feeding in home or community settings because our research question was about the early stage of enteral feeding in critically ill children that takes place while the child is in the hospital. Where studies looked at both home or community settings and hospital settings, we included these studies but only extracted data that described the hospital settings. We excluded studies that focused only on parenteral feeding, i.e., intravenous provision of nutrition.

We also excluded studies where the primary focus was on children with severe acute malnutrition (SAM) and anorexia since these children are already malnourished before they start enteral feeding, and we consider malnutrition an outcome of critical illness and nutritional support during this stage.

### Search methods for identification of studies

#### Electronic searches

The search strategy was developed by one of the review authors in consultation with an information specialist and the other review authors.

We searched the following electronic databases from their inception to July 2024 to identify eligible studies:

- MEDLINE via Ovid.
- CINAHL via EbscoHost.
- Embase via Ovid.
- Scopus via Elsevier.

We developed search strategies for each database. We did not apply any limits on publication date. Where applicable, we included a methodological filter for qualitative studies. See Additional file 1 for all the search strategies used.

Because of the limited time of the project, we did not search for grey literature.

#### Searching other resources

We reviewed the reference lists of all the included studies and key references (i.e., QES on related topics). The linked effectiveness systematic review of randomised controlled trials conducted as part of the GELA project had not started at the time of our search, so we could not check the effectiveness studies that were included in that review in our search for additional studies. Due to the time constraints of the project, we did not contact authors of included studies to clarify published information and to seek unpublished data.

### Selection of studies

Two review authors independently assessed the titles and abstracts of the identified records to evaluate eligibility. We retrieved the full texts of all papers identified as potentially relevant. Two review authors then assessed these papers independently. We resolved disagreements by discussion or, when required, by involving a third review author.

We have included a table listing studies that we excluded from our review at full text stage and the main reasons for exclusion (see table in Additional file 4).

We have included a PRISMA flow diagram (see figure in S1 figure) to show our search results and the process of screening and selecting studies for inclusion.

### Data extraction

We developed a data extraction form and extracted the following descriptive information:

- First author of the study, year of publication, country of study, study aim, study setting (type of hospital ward or department).
- Participants’ age, socioeconomic status, healthcare condition, cadre of health worker (e.g., nurse, doctor, dietitian, or other cadre identified as the healthcare worker),
- Type of enteral feeding (e.g., NGT, nasoduodenal tube, nasojejunal tube), type of feed (e.g., commercial feed, staple food, modified staple food), timing of feeding (early vs. delayed), who initiated the feeding (e.g. nurse, doctor, dietitian, parents).
- Country setting (e.g., high- or low‐/middle‐income country setting).
- Study design and sources of funding.

Secondly, we extracted all data relevant to the review aim. This included data describing the views, experiences, and behaviour of children, caregivers, healthcare workers and others involved in the provision of enteral feeding of hospitalised children.

At least one review author extracted data, with another author cross-checking to ensure that all relevant data has been extracted. Disagreements were resolved by discussion or in consultation with a third review author.

### Assessing the methodological limitations of included studies

One review author assessed methodological limitations for each study and another cross checked the assessments. We used a quality assessment tool for qualitative studies used in previous Cochrane Reviews [28, 29]. We resolved disagreements by discussion or, when required, by involving a third review author.

We reported our assessments in a Methodological Limitations table, using a ‘yes/no/partial’ rating and included explanations of any concerns we have, see Table 1. We used these assessments to support our GRADE-CERQual assessment of our confidence in the review findings [26].

### Data management, analysis and synthesis

We used a thematic synthesis method as our analytical approach [30]. One study author extracted the relevant study data from the included studies. A different author checked the extraction. The first author then coded the data in NVivo 14 using an inductive approach [30]. Before coding, all the extracted data were read through for familiarisation. Then, the extracted data from each study were coded line-by-line, beginning with the study that was most relevant to the research question. The initial codes were used to code subsequent studies, with new codes added to fit the data. Similar initial codes across studies were then grouped into more analytic codes, which were subsequently organised into broader themes. Review findings were then synthesised from the data that had been grouped into the same themes across the studies. Findings were shared with co-authors to review. Finally, we re-read the included studies to check that we had extracted all data relevant to the findings.

Once we finished preparing the review findings, we examined each finding, identifying factors that could influence the implementation of the intervention, and then developed prompts for future implementers. We shared this section with stakeholders involved in the wider GELA project to gather their feedback about the relevance of these prompts and the way they were phrased and presented.

### Assessing our confidence in the review findings

At least two review authors used the GRADE-CERQual approach to assess our confidence in each finding [26]. GRADE-CERQual evaluates the level of confidence in evidence by considering four main aspects: methodological limitations, coherence, adequacy, and relevance [26].

After assessing each of the four components, we made a judgement about the overall confidence in the evidence supporting the review finding, as required by GRADE-CERQual. We judged confidence as high, moderate, low, or very low. The final assessment was based on consensus among the review authors. All findings started as high confidence and were then graded down if there were important concerns regarding any of the four GRADE-CERQual components.

## Results

### Results of the search

**Fig. 1:**
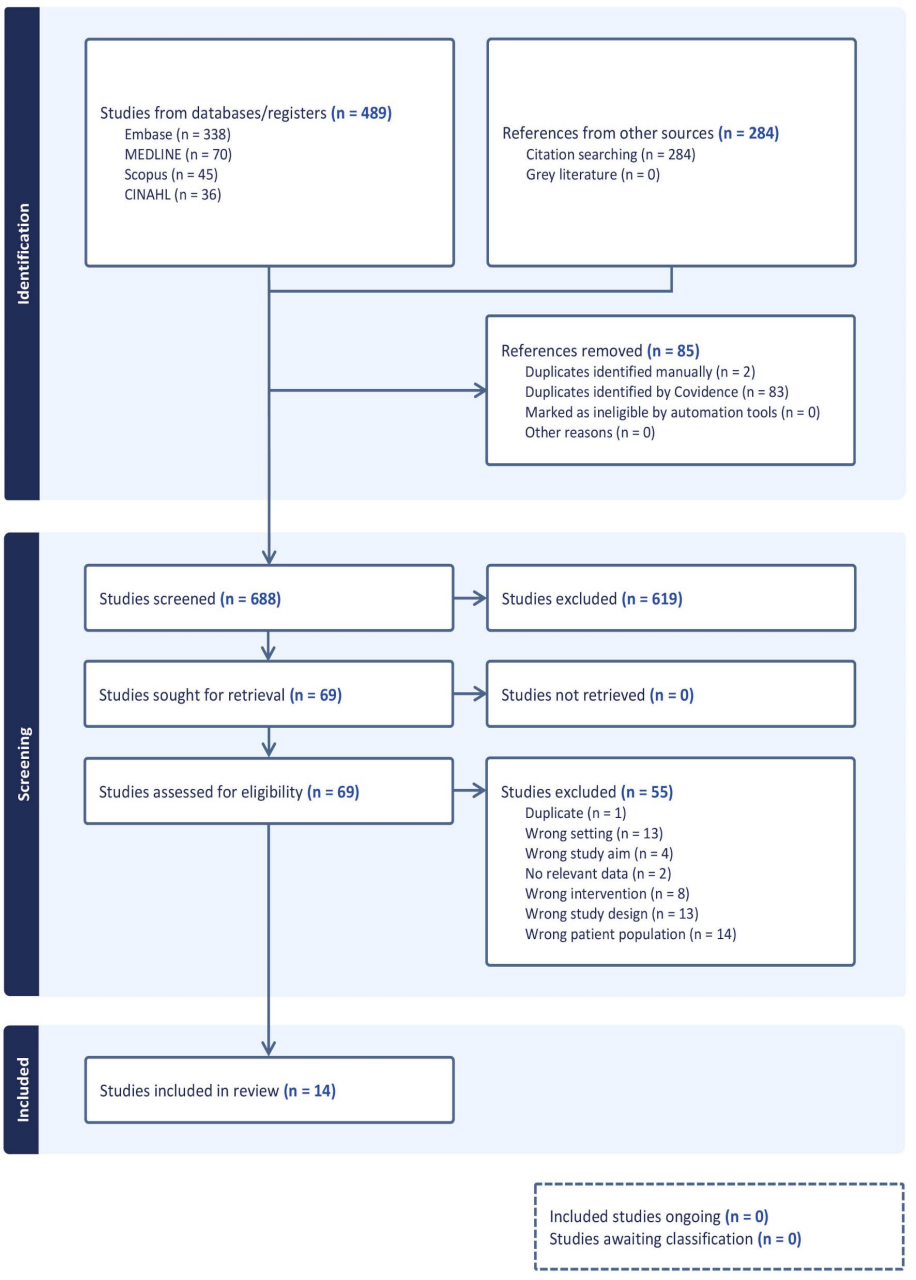
PRISMA study flow diagram

We included 14 studies in our review (see Fig. 1). These studies were published between 1985 and 2023.

### Description of the studies

See the Characteristics of Included Studies table in Additional file 5 for detailed descriptions of the included studies.

#### Study settings

Most studies (*n* = 9) were from high-income countries (HICs) [34–42], with two from low-income countries (Ghana [31], Malawi [32]) and three from upper-middle income countries (Brazil [43], Turkey [44], and South Africa [33]). Three studies were conducted in Africa: Ghana [31], Malawi [32], and South Africa [33]. Three studies were conducted in Europe: Switzerland [34] Sweden [35] and the Netherlands [36]. Three studies were done in Australia [37–39], three in North America (Canada [40] and USA [41, 42]), and one study each in South America (Brazil [43]), and Western Asia (Turkey [44]).

The included studies were carried out in different hospital settings. However, in two studies some, or all, of the participants were interviewed at home following discharge from hospital [35, 36]. Four studies were based in intensive care settings: PICU [34], NICU [35, 38] or both NICU and PICU [31]. Four studies were based in cancer wards or paediatric cancer wards [37, 41, 42, 44]. Two studies were conducted in general paediatric hospital wards [32, 40]. Three studies were based on more specialised hospital settings: a kangaroo mother care unit [33], an inpatient feeding therapy program [39], and a referral hospital for patients with craniofacial anomalies and related syndromes [43]. In one study, children had been admitted to hospital, but the type of hospital ward was unclear [36].

#### Participants

Studies sought the perspectives of parents, children and healthcare workers. Three studies included mothers only [33, 35, 43]. Two studies included mothers and fathers [38, 39]. One study included children only as participants [40]. Two studies included children and mothers or parents [41, 44]. Three studies included healthcare workers only, specifically nurses, physicians, and midwives [29, 31]. Two studies included parents and healthcare workers [36, 42]. One study included parents, children, and healthcare workers [37].

Three studies included children and adolescents up to the age of 18 as study participants [37, 41, 44]. The age ranges of the children participating in these studies were reported as: 11 months to 18 years [44]; under 18 years, mean 9,5 years, SD 5 years [36]. One of these studies described the age group they included as children from 6 years old to over the age of 18 [41], but we only included data on children under 18.

In six other studies, the children receiving enteral feeding were under the age of 10. The age ranges of the children in these studies were: 4 to 10 months [38]; 0 to 10 years [36]; 0 to 2 years [31]; and 2–6 years [40]. In two studies, the children were preterm or newborn infants [33, 35]. Five studies did not provide information about the age of the children receiving enteral feeding [32, 34, 39, 42, 43].

Two studies described the health condition of the children receiving enteral feeding as critical illness [31, 34]. Two studies focused on preterm or critically ill newborn infants [33, 35]. In four studies, the children had paediatric cancer [37, 41, 42, 44]. In three studies, the children had complex medical conditions including premature birth or congenital birth defects [38, 39, 43]. One study described children as having chronic illness [40]. In two studies, the children had multiple diagnoses, including both acute and chronic conditions [32, 36].

#### Route of enteral feeding

The main route of enteral feeding studied was the nasogastric route, although some studies also described other routes. Ten studies described experiences with NGT feeding [31, 32,35, 36, 38, 39, 40, 41, 43, 44]. In one study, most of the children had received NGT feeding and some children had received other enteral routes, but it was unclear which routes [37]. In one study, both gastric and postpyloric feeding was provided [34]. Some studies described experiences with parenteral nutrition [41, 42] or gastrostomy [32, 36] in addition to enteral feeding, but we only included the data from these studies that was relevant to our objective.

### Methodological limitations

Most studies were based on individual or focus group interviews, which we considered an appropriate method for eliciting views and experiences of enteral feeding (see Table 1 showing our assessment of methodological limitations). None of the studies that explored healthcare worker practices used observations, which could have more accurately captured healthcare workers’ practices.

Several studies had insufficient sampling strategies or insufficient reporting of the strategies used, which raised concerns about groups such non-English speakers or other minorities being left out of the samples.

Most of the studies showed poor reporting of reflexivity, and only one study showed any evidence of reflexivity. We found this to be a concern for several reasons. In some of the studies the researchers were clinicians in the hospitals where the studies were being carried out, and we were concerned about whether the informants’ responses may have been influenced by social acceptability bias. If informants had been in a patient relationship with the researchers, they may find it difficult to report negative perceptions. Further, two of the studies investigated interventions to improve clinical practices around enteral feeding. The study authors might have had interests in the outcomes of these interventions, which could have influenced the findings. For other studies we were concerned about how the researchers’ backgrounds may have influenced the design, conduct, or findings of the studies.

**Table 1:** Assessments of the methodological limitations of the studies

Author(s), Year	Were the settings and context described adequately?	Was the sampling strategy described, and was this appropriate?	Was the data collection strategy described and was this appropriate?	Was the data analysis described, and was this appropriate?	Were the claims made/findings supported by sufficient evidence?	Was there evidence of reflexivity?	Did the study demonstrate sensitivity to ethical concerns?	Any other concerns?
Abukari & Acheampong 2021	Yes	Yes	Partially	Yes	Yes	No	Yes	No
Banhara et al. 2020	Yes	Partially	Yes	Yes	Yes	No	Yes	No
Bicakli et al. 2019	Partially	Partially	Yes	Yes	Yes	No	Yes	No
Cohen et al. 2017	Partially	Partially	Yes	Yes	Yes	Yes	Yes	No
Daniel et al. 2019	Yes	Yes	Partially	Yes	Yes	No	Yes	No
Ellerton et al. 1985	Yes	Yes	Partially	Yes	No	No	Yes	No
Ferguson & Paul 2007	No	No	Yes	Yes	Yes	No	Yes	No
Lively et al. 2023	No	Partially	Partially	Yes	Partially	Yes	Yes	No
Madiba & Sengane 2021	Partially	Yes	Partially	Yes	Yes	No	Yes	No
Montgomery et al. 2013	Partially	Yes	Partially	No	Yes	No	Not clear	Yes
Moullet et al. 2020	Yes	Yes	Yes	Yes	Yes	Yes	Yes	No
Mӧrelius et al. 2020	Partially	Yes	Yes	Yes	Yes	No	Yes	No
Remijn et al. 2022	No	Partially	Yes	Yes	Yes	No	Yes	No
Williams-Hooker et al. 2015	Partially	Not clear	Partially	Yes	No	No	Yes	No

### Confidence in the review findings

Using the GRADE-CERQual approach, we assessed five findings as moderate confidence, and eight as low confidence, and one as very low confidence (see the Summary of Qualitative Findings table in Table 2). Our explanation of the GRADE-CERQual assessment for each review finding is shown in the full evidence profiles (see Evidence Profile table in Additional file 6).

**Table 2:** Summary of qualitative findings (SoQF)

#	Summarised review finding	GRADE-CERQual Assessment of confidence	Explanation of GRADE-CERQual Assessment	References
1	Parents and older or adolescent children described being worried about discomfort, pain, and other complications of tube feeding before enteral feeding was initiated. Their anxiety appeared to be related to a lack of knowledge and experience of enteral feeding.	Moderate confidence	Minor concerns regarding adequacy because of thin data. Moderate concerns regarding relevance because of indirect data (from non-intensive care settings). No or very minor concerns regarding methodological limitations and coherence.	Montgomery et al. 2013; Williams-Hooker et al. 2015; Ferguson & Paul 2007; Banhara et al. 2020; Cohen et al. 2017; Bicakli et al. 2019; Madiba & Sengane 2021; Daniel et al. 2019;
2	Where parents were present during nasogastric tube insertion, some found the procedure difficult to watch because of the discomfort they observed their child experiencing. Other parents focused on how the discomfort did not last long and on the fact that the child recovered quickly.	Moderate confidence	Moderate concerns regarding adequacy because of thin data from few studies. Minor concerns regarding relevance because of indirect data (from non-intensive care settings). No or very minor concerns regarding methodological limitations and coherence.	Ferguson & Paul 2007; Cohen et al. 2017; Bicakli et al. 2019; Madiba & Sengane 2021;
3	Some parents felt more positive about nasogastric tube feeding after the tube had been inserted. Some reported that their children seemed to get used to the tube easily, while others reported seeing benefits of enteral feeding such as weight gain, better nutrition, easier administration of medication, and a less stressful feeding situation. Some older children and adolescents appeared to agree, although they expressed more ambivalent or negative feelings about the tube. Other parents and older children also described specific challenges during tube feeding such as vomiting, diarrhoea or other kinds of discomfort, and parents described how these challenges caused new concerns.	Moderate confidence	Moderate concerns regarding adequacy becuase of thin data from few studies. No or very minor concerns regarding methodological limitations, coherence, and relevance.	Mӧrelius et al. 2020; Montgomery et al. 2013; Remijn et al. 2022; Ferguson & Paul 2007; Banhara et al. 2020; Cohen et al. 2017; Bicakli et al. 2019; Ellerton et al. 1985; Madiba & Sengane 2021; Lively et al. 2023;
4	Before the nasogastric tube was inserted, some children and parents expressed concern about what the tube would look like. After insertion, some parents described getting used to seeing their child with the tube, although some older children and adolescents continued to feel self-conscious about their appearance.	Very low confidence	Serious concerns regarding adequacy because of limited and very thin data (3 studies). Minor concerns regarding methodological limitations because of inadequate sampling and reporting of sampling and reflexivity. Moderate concerns regarding relevance because of indirectness (data from non-intensive care settings). No or very minor concerns regarding coherence.	Ferguson & Paul 2007; Cohen et al. 2017; Bicakli et al. 2019;
5	Mothers of preterm and critically ill newborns described tube feeding as emotionally challenging. These mothers said they felt frustrated when they could not breastfeed, and they reported feeling that tube feeding disrupted bonding and attachment. Some mothers felt excluded and powerless when healthcare workers took over the care of their infants, but they also reported needing support from healthcare workers as well as partners and others. Many mothers described transitioning from tube feeding to breastfeeding as a positive experience, but some mothers were concerned about weight loss due to the infant’s poor suckling abilities.	Low confidence	Serious concerns regarding adequacy because of thin data from few studies. Minor concerns regarding relevance because the finding may not be relevant in all settings. No or very minor concerns regarding methodological limitations and coherence.	Mӧrelius et al. 2020; Banhara et al. 2020; Cohen et al. 2017; Madiba & Sengane 2021;
6	Healthcare workers and mothers of preterm and critically ill newborns described how expressing breast milk and sustaining milk supply while the infant was tube feeding was difficult. Although mothers were highly motivated to provide breastmilk for their infant, they said they needed support from healthcare workers, partners and other parents to succeed.	Moderate confidence	Moderate concerns regarding adequacy because of few studies. No or very minor concerns regarding methodological limitations, coherence, and relevance.	Mӧrelius et al. 2020; Abukari & Acheampong 2021; Madiba & Sengane 2021;
7	While some parents described a sense of participation in the decision-making process on whether to initiate enteral feeding for their child, others described how they were not involved in decisions related to this. Some healthcare workers agreed that parents should be more involved.	Low confidence	Serious concerns regarding adequacy because of few studies and very thin data. No or very minor concerns regarding methodological limitations, coherence, and relevance.	Mӧrelius et al. 2020; Remijn et al. 2022; Ferguson & Paul 2007; Cohen et al. 2017; Daniel et al. 2019;
8	Some parents described how the hospital environment offered opportunities to exchange support with parents in similar situations and could help normalise the process. Other parents felt that the lack of support from staff made the hospital a difficult environment.	Low confidence	Serious concerns regarding adequacy because of very thin data from few studies. No or very minor concerns regarding methodological limitations, coherence, adequacy, and relevance.	Mӧrelius et al. 2020; Remijn et al. 2022; Ferguson & Paul 2007;
9	Some parents reported receiving information about enteral feeding from hospital staff, while other parents said the information they had been given was not enough. Parents’ perception of the adequacy of the information they receive could be related to the way the information was conveyed. A few parents sought information from other sources than healthcare workers, including from other families.	Low confidence	Serious concerns regarding adequacy because of thin data and few studies. No or very minor concerns regarding methodological limitations, coherence, adequacy, and relevance.	Mӧrelius et al. 2020; Remijn et al. 2022; Ferguson & Paul 2007; Cohen et al. 2017;
10	Some parents said they would have wanted some types of information at earlier stages, for example about duration and consequences of enteral feeding. Healthcare workers agreed that parents needed adequate information at each stage of enteral feeding, but both parents and healthcare workers reported that parents might not remember information given at early and stressful stages of their child’s illness.	Low confidence	Serious concerns regarding adequacy because of thin data from few studies. No or very minor concerns regarding methodological limitations, coherence, and relevance.	Remijn et al. 2022; Ferguson & Paul 2007; Cohen et al. 2017;
11	Some healthcare workers reported that they lacked knowledge and skills to provide adequate enteral feeding for critically ill children. They described variations in practice due to a lack of clear criteria for initiation. Some healthcare workers who had received some training in nutrition support suggested that the training was insufficient.	Low confidence	Serious concerns regarding adequacy because of thin data from few studies. No or very minor concerns regarding methodological limitations, coherence, and relevance	Abukari & Acheampong 2021; Cohen et al. 2017; Moullet et al. 2020;
12	Healthcare workers described the main benefits of enteral feeding as ensuring adequate nutrition and weight gain or maintenance. Some healthcare workers also suggested that enteral feeding could lead to better health outcomes and reduced risk of death; shorter hospital stays and reduced risks of readmission; and improved tolerance among children of clinical interventions. Healthcare workers also believed that enteral feeding not only made it easier to feed the child but also to give medication. Healthcare workers described potential negative consequences of tube feeding as including vomiting, diarrhoea, abdominal pain, and the risk of aspiration. Some healthcare workers were concerned about the discomfort for the child as well as parents’ concerns about these negative consequences.	Low confidence	Serious concerns regarding adequacy because of thin data from few studies. No or very minor concerns regarding methodological limitations, coherence, and relevance.	Abukari & Acheampong 2021; Williams-Hooker et al. 2015; Cohen et al. 2017; Daniel et al. 2019;
13	Some healthcare workers reported that the introduction of nutritional tools, protocols or other intervention to improve practices could increase their knowledge and awareness of nutrition support, although a lack of time could be a barrier to using new tools. Some healthcare workers also emphasised the importance of including dietitians in care as this could be a valuable source of guidance, could improve nutritional practices, and could reduce their workload.	Low confidence	Serious concerns regarding adequacy because of thin data from few studies. Minor concerns regarding methodological limitations because study authors’ lack of reflexivity may have influenced the design and conduct of the studies. No or very minor concerns regarding coherence and relevance.	Abukari & Acheampong 2021; Cohen et al. 2017; Moullet et al. 2020; Daniel et al. 2019;
14	Some healthcare workers reported that a lack of resources could be a barrier to providing adequate nutritional support, for example when nutritional support was not consistently available or primarily made available to malnourished children, or where storage facilities for breast milk were lacking.	Moderate confidence	Moderate concerns regarding adequacy because of thin data from few studies. No or very minor concerns regarding methodological limitations, coherence, adequacy, and relevance.	Abukari & Acheampong 2021; Cohen et al. 2017; Daniel et al. 2019;

Our main concerns were related to the relevance of the supporting studies and the adequacy of the data. For several findings, the data were either indirect or unclear regarding the setting or intervention. In addition, many of the findings were supported by few studies and thin data. By thin data, we mean data that does not provide sufficient detail to enable us to understand the phenomenon described in a finding [45].

### Review findings

We organised our 14 findings into three categories. In the first category, we present findings that are related to parents’ and children’s experiences and perceptions of receiving enteral feeding. In the second category, we focused on findings about parents’ experiences of interacting with healthcare workers and other parents in the hospital setting, including participating in decision-making and receiving information and support. Since we had found few studies from intensive care settings, we primarily relied on indirect evidence to support the findings in these two categories. Nevertheless, based on our discussions with stakeholders and within the review team, we have considered that the experiences and perceptions described by parents and children in these studies can apply to intensive care settings. However, the indirectness of the data has contributed to the moderate and very low confidence ratings we have for these findings. The third category presents findings about healthcare workers’ experiences and perceptions of providing enteral feeding to critically ill children. Studies from the PICU setting contribute to these four findings. Although these studies provided relevant data to the intensive care setting, we were concerned about the adequacy of the data due to the limited number of studies and thin data. The findings are also in part supported by studies from other settings, including NICU settings. Still, for these findings, we had less concern about indirect relevance as we considered the data to be applicable to PICU settings. Consequently, our low confidence in these findings was predominantly related to the adequacy of the data.

See the summary of qualitative findings (SoQF) (see Table 2) for references to the studies contributing data to each finding and an explanation of our GRADE-CERQual assessment of confidence each finding [24]. All review findings that were assessed using GRADE-CERQual are reported in the SoQF table regardless of their associated level of confidence. We present detailed descriptions of our confidence assessment in the GRADE-CERQual evidence profiles (see the table in Additional file 6).

#### Category 1: parents’ and children’s experiences and perceptions of receiving enteral feeding

**Finding 1: Parents and older children described being worried about discomfort**,** pain**,** and other complications of tube feeding before enteral feeding was initiated. Their anxiety appeared to be related to a lack of knowledge about and experience of enteral feeding (moderate confidence).**

Parents in many studies from non-acute settings described concerns and fears that parents had about tube feeding before feeding was initiated [32, 33, 37, 41–44]. One study reported that parents’ perceptions of enteral feeding as painful and invasive could be a barrier to their acceptance of the intervention [37]. These studies were mostly from non-intensive care settings.

The most commonly described concerns reported by parents were fears that the intervention would cause pain or discomfort for the child [33, 37, 41–44]. Some parents were concerned about how the child’s skin would react to having the tube taped onto their face [37]. The concern about tube insertion causing pain for the child was also a source of worry:

“*Yho! Feeding a baby with a tube is scary because you sometimes wonder if it doesn’t hurt her when they put it inside of her. (25-year-old mother)”* [33].

Related to concerns about discomfort, parents also reported that they were concerned about the invasiveness of the intervention [37, 42]. Some parents were concerned about how the child would cope with the intervention [37]. Some studies reported that parents associated tube feeding with risk of death [32, 43], and healthcare workers reported that parents may refuse the intervention because of this association [32]. Parents also worried about the negative long-term consequences of enteral feeding, such as the children not growing or developing normally if tube fed [44]. On the other hand, parents were also worried that their children could die or not recover from illness if they did not receive nutrition [44].

Some children were also concerned about discomfort and feared the procedure before the tube was inserted [37, 41, 44]:

*Don’t want things down my nose or throat. (13-year-old female with history of Burkitt’s lymphoma)* [41].

*Tube feeding sounds disgusting and uncomfortable. (15-year-old female with history of ALL)* [41] (The article does not state what ALL is, but we assume it to be acute lymphoblastic leukaemia).

Some studies suggested that parents’ anxieties and fears about enteral feeding could be related to their lack of knowledge. Some parents described the possibility of complications that could arise or uncertainty about outcomes as contributing to their concerns about the intervention [43, 44]. Two studies reported that parents associated the intervention with death [32, 43], and healthcare workers in one of these studies suggested that parents might refuse the intervention because of the this fear. Some children and parents described the uncertainty about the insertion procedure as a cause of fear [43, 44]:

*I felt nervous at first; to be frank*,* I was scared*,* probably because I did not know what it was and what it would be like (female*,* age 18*,* nasopharynx cancer).* [44]

Several studies reported that parents lacked prior knowledge and experience of enteral feeding [33, 36, 38, 42]. Most parents and caregivers did not have prior experience of enteral feeding, and only some caregivers had knowledge about enteral feeding [33, 38, 42]. One study reported that the lack of knowledge could contribute to feelings of being overwhelmed, emphasising the link between parents’ lack of knowledge and their fears before feeding initiation:

*“We found that one of the feeding issues that generated the most fear*,* stress*,* and uncertainty among the mothers was the lack of knowledge concerning the tube feeding of infants during admission in NICU.”* [33].

Another study quoted a mother who perceived enteral feeding as “unnatural”, which could suggest a lack of knowledge about nutrition during illness:

*‘It are just my own gut feeling that there must be a reason why when we are sick we don’t want to eat.… but*,* wouldn’t it make sense that if our body doesn’t want to eat that we don’t need food?’* [37].

**Finding 2. Where parents were present during nasogastric tube insertion**,** some found the procedure difficult to watch because of the discomfort they observed their child experiencing. Other parents focused on how the discomfort did not last long and on the fact that the child recovered quickly (moderate confidence).**

In studies from non-intensive care settings, some parents described how seeing the nasogastric tube inserted into their child is a difficult emotional experience because of the pain and discomfort the procedure caused the child [33, 37, 38]. In one study a mother reported that she insisted on general anaesthetic for her child to make the tube insertion procedure less painful [37]. One study reported that healthcare workers agreed that the insertion procedure can be a negative or traumatic experience for parents [37]. Some parents reported feeling bad about contributing to causing their child pain and discomfort:

*‘I burst into tears I couldn’t believe I had just done that to my son.’* [38].

For other parents, seeing the tube inserted into the child’s body seemed to cause them distress:

*‘She was little at that stage*,* to see something like that go into her body was horrible you know.’* [38].

However, not all parents seemed to experience the insertion process as traumatic. Some parents focused on how the child recovered quickly from the discomfort of putting in the tube [37, 38, 44]. Some parents who inserted the tube themselves described switching off to their child’s pain [38]. Other parents who found their child’s distress uncomfortable to witness, saw that the pain or discomfort did not last long and that the child felt better once the tube was inserted:

*‘Every time [the patient] cried*,* [when] they tried to put in [the tube]*,* but after that she was fine again’ (Mother: female*,* Wilm’s Tumour*,* eight years).* [37]

*‘He suffered only once when the tube was inserted. Otherwise*,* we have to try to force-feed him four or five times a day every day. He will hate eating. Tube insertion is a momentary pain; it takes only 30 seconds.’* [44].

*‘She’s a very forgiving little person. Often after she been screaming*,* having the tube done. She looks up as though to say “Well. Thank You”. she seems to know that it’s necessary*,* that it’s not a deliberate ploy on my part to torture her.’* [38].

**Finding 3: Some parents felt more positive about tube feeding after the tube had been inserted. Some reported that their children seemed to get used to the tube easily**,** while others reported seeing benefits of enteral feeding such as weight gain**,** better nutrition**,** easier administration of medication**,** and less stress. Some older children and adolescents appeared to agree**,** although they expressed more ambivalent or negative feelings about the tube. Other parents and older children also described specific challenges during tube feeding such as vomiting**,** diarrhoea or other kinds of discomfort**,** and parents described how these challenges caused new concerns (moderate confidence).**

In some studies from non-acute settings, parents described how they and their child got used to tube feeding [33, 35, 38]. Mothers of preterm babies, who tended to be more negative about tube feeding generally due to their frustration at being unable to breastfeed their babies, stated that they got used to feeding their child with a tube [33, 35]. Some parents described their babies as unconcerned about the tube, or at least accepting the tube after the discomfort of insertion is over.

*‘She loves the tube. She plays with it.’* [38].

Parents in several studies described how they felt more positive about tube feeding after the tube had been inserted because they could see the benefits of enteral feeding [33, 35, 37–39, 43, 44]. Seeing the benefits of enteral feeding for their child allowed parents to understand the necessity of enteral feeding [33, 38, 39]. Psychological benefits for parents included knowing that the child was well-nourished and less worry about nourishment. When children had severe eating difficulties, parents also viewed the tube as life-saving [43].

Parents also reported other practical aspects of tube feeding as benefits of tube feeding. Some parents viewed the opportunity of feeding the child when sleeping as a benefit [33, 37]. In the studies of paediatric oncology patients, some parents and healthcare workers said they appreciated that NG tube feeding made it easier to give children medication, sedatives or nutritional supplements [37, 41, 44]. Some studies reported that parents would recommend tube feeding to other parents based on their experience of the benefits [37, 44].

For parents of paediatric cancer patients and preterm babies, weight gain was a main positive factor [33, 37, 44]. Weight gain was significant for mothers of preterm babies since sufficient weight gain was a criterion for being allowed to go home from the hospital [33].

Some children also described seeing the benefits of weight gain, even when they had more negative perceptions of the tube [37, 44]:

*‘I really hate it to be honest but I do know that right now*,* it’s the only way I can really gain weight’ (Patient: male*,* MDS*,* 13 years).* [37]

Children seemed to have more ambivalent experiences of tube feeding after the tube had been inserted. Of the studies that described children’s experiences with tube feeding, only two studies included any data from children’s perspectives about how they experienced tube insertion or having the tube in their noses [37, 40]. One study included young children as participants by asking them to play out intrusive hospital procedures. This study suggested that the children who had experienced nasogastric feeding did not seem to display much concern about tube feeding [40]. Other children seemed to have more ambivalent perceptions of tube feeding. One study suggested that parents believed that their babies who were tube-fed had “a love-hate relationship to the tube” [38]. One of the studies of paediatric cancer patients suggested that enteral feeding made children feel more sick:

*‘I’ve always felt sick and I’ve always felt worse having the feeds continuous’ (Patient: male*,* MDS*,* 17 years).* [37]

In many studies, parents described challenges that could arise during tube feeding which gave them new reasons for concern [33, 35–39, 43, 44]. Some parents described ambivalent feelings about the tube and the care provided, expressing both anger and gratitude towards healthcare workers [38].

Problems that arose during tube feeding that caused parents concern included the child vomiting up the tube [36, 37] pulling out the tube [33], diarrhoea [37], the tube blocking [37] or the tube or tape on the face causing discomfort [33, 37, 41].

Some parents reported concerns about oral eating during enteral feeding or the return to oral eating [36, 37]. For instance, some parents described concerns that their child stopped eating even though it was recommended that they eat orally in addition to the tube feeding [36, 37]. One healthcare worker suggested that eating orally when a nasogastric tube was inserted was uncomfortable so this could be one reason why children would stop eating completely. If the child stopped all oral eating, some parents became concerned about long-term impacts on feeding [36, 37].

One child described sleep deprivation as a negative consequence of enteral feeding due to the disruption by the machines:

*‘During the night*,* the machine’s always turning and it constantly makes noise and when it runs out it beeps and everyone has to get up and fix it’ (Patient*,* female*,* medulloblastoma*,* 17 years).* [37]

**Finding 4: Before the nasogastric tube was inserted**,** some children and parents expressed concern about what the tube would look like. After insertion**,** some parents described getting used to seeing their child with the tube**,** although some older children and adolescents continued to feel self-conscious about their appearance (very low confidence).**

Before tube insertion, some children and parents in studies from non-intensive care settings reported being concerned about how the tube would look on their face [37, 44]. After insertion, some parents reported that they got used to seeing their baby with the tube. In one study, parents described seeing the tube as part of the baby:

*‘It’s part of her. If she hasn’t got it in I think she looks so strange.’* [38].

Children’s concerns about the appearance of the tube seemed to continue after insertion [37, 44]. Parents and healthcare workers reported that some children were self-conscious and reluctant to be seen by others in the hospital [44].

*‘I didn’t particularly like the way that it identifies you as a sick person’ (Patient: male*,* 17 years*,* Biphenotypic Leukaemia).* [37]

**Finding 5: Mothers of preterm and critically ill newborns described tube feeding as emotionally challenging. These mothers felt frustrated when they could not breastfeed. These mothers felt that tube feeding disrupted bonding and attachment. Some mothers felt excluded and powerless when the care of their babies was taken over by healthcare workers**,** but they also reported needing support from healthcare workers as well as partners and others. Many mothers described transitioning from tube feeding to breastfeeding as a positive experience**,** but some mothers were concerned about weight loss due to the baby’s poor suckling abilities (low confidence).**

Studies that focused on tube feeding in preterm and critically ill children and newborns suggested that these mothers experienced tube feeding as emotionally difficult [33, 35, 43]. These mothers were already concerned about the health of their children who were extremely vulnerable, and even though they appreciated that the infants were getting the nutrition they needed, they seemed to experience tube feeding more negatively than parents of other children [33, 35]. Some mothers worried that tube feeding could hurt their baby and said they did not get used to tube feeding over time [33].

Part of the difficulty seemed to be related to the mothers’ frustration at not being able to breastfeed [33, 35, 37, 43]. Mothers expected to breastfeed their children and were disappointed when breastfeeding was not possible [35, 37, 43]. Some mothers felt that breastfeeding was expected of them and that it would be the best for their child, so not being able to breastfeed threatened their role as mothers [35].

Mothers also appeared to experience the loss of breastfeeding as interfering with their ability to bond and form attachment with their child [33, 35]. Intermittent rather than continuous tube feeding seemed to be preferred by some mothers as it allowed for feeding situations throughout the day that offered more opportunities for bonding [35].

Having many other people involved in the care of their child could also contribute to difficulties in bonding or feelings of exclusion and powerlessness [35]. Some mothers appeared to feel their role as a mother was taken away from them due to the baby’s dependence on machines and healthcare workers [35]. One study suggested that the psychological impact of having an infant whose feeding was interrupted negatively impacted the parents’ quality of life [43]. For some mothers, not being able to make decisions for their child in the way other parents could further add to feelings of loss of control [35]. However, when mothers were asked to tube feed the infant themselves, some mothers seemed to find this to be an overwhelming additional burden in a an already stressful situation:

*Tube feeding stressed me because I thought the doctors and nurses were the ones who were supposed to do it. So*,* when I realised that I had to tube feed my baby myself*,* I was stressed. The thing is there were too many things to deal with and to add to that*,* I had to tube feed my baby. It was depressing*,* seriously. (22-year-old mother)* [33].

Some mothers of preterm infants reported that support from healthcare workers, partners and other mothers was very important to them in their struggle [33, 35].

Some mothers described the transition from tube feeding to breastfeeding as a pleasurable and rewarding experience [33, 35]. However, some mothers were concerned about the baby losing weight when because they were not able to suck well and it could be difficult to tell how much milk they were getting [33].

Some mothers described the transition from tube feeding to breastfeeding as a pleasurable and rewarding experience [33, 35]. However, some mothers were concerned about the baby losing weight when because they were not able to suck well and it could be difficult to tell how much milk they were getting [33].

#### Category 2: Parents’ experiences and perceptions of communication, information and support related to their child’s enteral feeding

**Finding 6: Healthcare workers and mothers of preterm and critically ill newborns described how expressing breast milk and sustaining milk supply while the infant was being tube fed was difficult. Although mothers were highly motivated to provide breastmilk for their infant**,** they said they needed support from healthcare workers**,** partners and other parents to succeed (moderate confidence).**

Some mothers of preterm and critically ill newborns described expressing breastmilk for enteral feeding as a stressful and painful process [33, 35]. Some mothers described how expressing milk using a pump caused exhaustion and breast pain [33]. Some mothers described the lack of privacy afforded by the hospital environment as a further difficulty [35]. Even when mothers were motivated to provide breastmilk for their babies, some mothers and healthcare workers reported that sustaining the milk supply could be difficult because of stress [31, 33, 35]. Not expressing enough milk then added to the stress and frustration experienced by the mothers [33, 35], but some mothers felt encouraged by being able to express even a small amount [33]. Some mothers felt unsupported by healthcare workers when their efforts to express milk were not appreciated by staff and expressed that they needed support from staff, other parents, and partners [35].

**Finding 7: While some parents noted that they felt they had participated in the decision-making process on whether to initiate enteral feeding for their child**,** others described how they were not involved in decisions related to this. Some healthcare workers agreed that parents should be more involved (low confidence).**

Some parents described how they were not included in the decisions around their child’s enteral feeding [35–37]. Some parents expressed that they would have preferred more specific criteria for initiation [37]. Some parents described their experiences of interactions with healthcare workers in negative terms:

*‘They felt powerless*,* unlistened to and*,* with their baby*,* at the mercy of the system*.’ [38].

One study reported that parents felt overlooked when healthcare workers followed protocols without taking parents’ concerns about the child’s reactions such as vomiting into consideration:

*‘While parents felt that they knew their child best and had a basic instinct for what was good or not for their child*,* they felt that HCPs sometimes maintained their protocols and barely listened to them or paid attention to the individual child’s needs. “The healthcare workers just followed the schedule*,* while we saw that she was reacting very badly to it. She vomited a lot. When I said*,* “Stop now*,* because she’s only vomiting”*,* they looked at me like*,* “Yes*,* but the schedule indicates something different. She must be fed now. Why are you meddling with that?” (I6*,* mother of a two-and-a-half-year-old girl).’* [36].

Parents also felt powerless when healthcare workers made changes to the child’s feeding methods without consulting them. For example, a mother experienced a lack of control when her breastmilk supply was deemed to be insufficient and staff began feeding with formula without consulting the mother [35]. One study reported that some parents found it difficult to question healthcare workers’ decisions unless they already had some healthcare knowledge, for example due to having a healthcare profession [36].

In one study some parents described feeling that they participated [35]:

*We’ve always felt that we’ve participated*,* but needed to put our foot down and show that it was us who steered the ship. You don’t want to feel as though just a passenger either; it’s after all our decision*.

Some healthcare workers agreed that parents should be more involved in decision-making and care [32, 36]. In one study, a panel of experts suggested that a decision-making tool could help healthcare workers involve parents in decision-making [36]. In another study, healthcare workers described the involvement of parents into care as a benefit [32].

**Finding 8: Some parents described how the hospital environment offered opportunities to exchange support with parents in similar situations and could help normalise the process. Other parents felt that the lack of support from staff made the hospital a difficult environment (low confidence).**

Some parents described certain aspects of the hospital environment as a source of social support [35, 36, 38]. Parents in one study reported that the hospital environment allowed parents to share difficult feelings with healthcare workers and other parents in similar circumstances [36]. Similarly, in another study, mothers expressed that they also experienced providing support to other parents as positive [35]. Some parents felt comforted by seeing other children who were also being tube fed because this normalised the process [38]. However, other parents described the hospital environment as a difficult environment where they had to learn the rules in order to cope:

*‘With my sister*,* both her and I were reprimanded for looking at the other babies and thought “Goodness*,* what have we gotten into here?”. When we found out what the rules are we thought “”Oh well. We’ll fit in here”.’* [38].

**Finding 9: Some parents reported receiving information about enteral feeding from hospital staff**,** while other parents said the information they had been given was not enough. Parents’ perceptions of the adequacy of the information they receive seem**,** in some situations**,** to be related to the way the information was conveyed. A few parents sought information from other sources than healthcare workers**,** including from other families and the internet (low confidence).**

Some parents reported not receiving enough information about enteral feeding from hospital staff [35–37], but others felt they had received adequate information [36]. One study found that most parents reported receiving limited information about the choice and necessity of tube feeding, and no information about possible adverse reactions from the child such as vomiting up the tube [36]. One study reported that some parents said they had not received any information before enteral feeding was initiated:

*‘But some parents were not informed about the start of tube feeding during in-patient care. “She was tube fed for the first time on the fourth day of hospitalisation. We were not really informed about this. In the beginning*,* it was constant tube feeding…. But I always ask about how much she is getting and why. Actually*,* they didn’t tell me much about it. I had to ask.” (Interview (I) 1*,* mother of an 8-month-old girl) Another mother had a similar experience: “I don’t think it was even communicated; they just put the nasal tube in. They didn’t say she was going to get a tube.” (I 9*,* mother of a 6-year-old girl).’* [36].

Whether parents perceived the information as adequate could also be related to the way information was given by healthcare workers. For the mothers of preterm infants, the perceived lack of information was perceived as a source of wear and tear on mothers [35]. These mothers reported that they wanted information to be given “in a neutral tone without being criticised” [35].

Two studies mention parents seeking information from other sources than healthcare workers [35, 37]. One parent of a child with a cancer diagnosis sought information about enteral feeding from other families [37]. Some mothers who said they did not receive adequate information about breastfeeding when their preterm baby was being fed enterally sought information about breastfeeding on the internet [35].

**Finding 10: Some parents said they would have wanted some types of information at earlier stages**,** for example about the duration and consequences of enteral feeding. Healthcare workers agreed that parents needed adequate information at each stage of enteral feeding**,** but both parents and healthcare workers reported that parents might not remember information given at early and stressful stages of their child’s illness (low confidence).**

Some studies emphasised that parents wanted information about the process and longer-term consequences of enteral feeding at an earlier stage [36–38]. Parents reported that they had received very little information about the duration and consequences of enteral feeding [36]. Parents whose children needed to continue tube feeding after discharge from the hospital felt they did not receive enough information about how to how to continue feeding at home [38]. Healthcare workers agreed that parents should receive appropriate information at the different stages of the enteral feeding process [36]. However, healthcare workers appeared to be hesitant about giving information about long-term consequences at early stages:

*‘A pediatrician added: ‘If a child is very ill in the ICU and receives tube feeding*,* it is usually not (yet) the right time to talk to parents about the consequences of tube feeding. The parents are then mainly focused on the survival of their child. As soon as the life-threatening situation is over or if the child goes home and continues tube feeding*,* it is a good time to inform the parents about the consequences of tube feeding.”’* [36].

For the children with cancer diagnoses, it appeared that healthcare workers did not generally provide information about the possibility of tube feeding during treatment at the time of diagnosis. Parents who were interviewed after their children had been tube fed were uncertain about whether they had received information at diagnosis or not. They seemed to agree that it was unlikely that they would remember information if it had been given at the time of diagnosis due to the stress of receiving a cancer diagnosis [37]. It is unclear whether these parents perceived the information they had received as inadequate, but the data seems to suggest that it is likely that parents will forget information given at the time of diagnosis. However, it is unclear how and when the parents of these children received information about enteral feeding at a later point and whether they found that information adequate.

#### Category 3: healthcare workers’ experiences and perceptions of providing enteral feeding for critically ill children

**Finding 11: Some healthcare workers reported that they lacked knowledge and skills to provide adequate enteral feeding for critically ill children. They described variations in practice due to a lack of clear criteria. Some healthcare workers who had received some training in nutrition support suggested that the training was insufficient (low confidence).**

Some healthcare workers in NICUs or PICUs described how they lacked knowledge and skills for enteral feeding [31, 34]. In one study, healthcare workers described their practices as imprecise, variable, and lacking fixed rules [34]. For example, physicians in a PICU described being unsure about caloric requirements for critically ill children, and they did not calculate energy or protein requirements during the PICU stay [34]. They also said they lacked knowledge about the introduction and increase of enteral nutrition as well as nutrition support after extubation [34].

Healthcare workers in one study described how some staff would not feed critically ill children because they lacked confidence in their skills:

*‘Some reported that*,* due to inadequate knowledge and skill*,* some clinicians will not feed the babies if they did not feel confident and skilful for fear of aspiration and death as recounted by a neonatal nurse: “For feeding in the intensive care unit*,* there is the need for requisite knowledge and being careful in order to avoid aspiration. So*,* for some of the staff*,* in order not to expose themselves*,* they leave the breast milk untouched without attempting to feed the babies as several instances have occurred on this ward where feeding of the babies led to deaths. This is because aspiration in preterm babies is very dangerous and most staff too working here have not specialized in intensive care and care of neonates” TSD1A’* [31].

Junior PICU physicians in one study said they based their practices on senior physicians’ knowledge and practices, which seemed to vary from case to case and not follow any specific rules [34]. In another study, healthcare workers said they relied on the dietitians’ advice on when to initiate feeding and emphasised the need for clearer criteria for when to initiate enteral feeding [37].

In one study, some healthcare workers mentioned having received some training in nutrition support, but it was unclear whether they found this training to be sufficient [34]. Junior physicians in this study who had received a “a 2-h introductory course” seemed to use notes from this course as their primary reference tool in the PICU but still said they lacked knowledge about nutrition [34].

**Finding 12: Healthcare workers described the main benefits of enteral feeding as ensuring adequate nutrition and weight gain or maintenance. Some healthcare workers also suggested that enteral feeding could lead to better health outcomes and reduced risk of death; shorter hospital stays and reduced risks of readmission; and improved tolerance among children of clinical interventions. Healthcare workers also believed that enteral feeding not only made it easier to feed the child but also to give medication. Healthcare workers described potential negative consequences of tube feeding as including vomiting**,** diarrhoea**,** abdominal pain**,** and the risk of aspiration. Some healthcare workers were concerned about the discomfort for the child as well as parents’ concerns about these issues (low confidence).**

Healthcare workers agreed with parents that the main benefit of enteral feeding was ensuring nutrition for the child [37, 42]. In one study, healthcare workers in the ICU reported that patients who had benefitted from nutrition support in their hospital were “*children with burns*,* typhoid perforations*,* hydrocephalus*,* pneumonia*,* acute malnutrition*,* cerebral palsy (CP)*,* and cancer*” [32]. Healthcare workers also described weight gain or maintenance as an important benefit [32, 37]. Healthcare workers described observing benefits such as quicker recovery, reduced risk of death and readmission, and increased tolerability of treatments in both critically ill children and cancer patients [32]. Other benefits they described were ease of medication and less conflicts about parents and children around eating. Some healthcare workers also emphasised the tolerability of the tube as an advantage [37].

*‘One of the big advantages is once you get the tube in it is well tolerated*,* once you get past the first couple of days and it is not uncomfortable anymore’ (Consultant).* [37]

When comparing enteral nutrition with parenteral nutrition, healthcare providers for paediatric oncology patients said they viewed enteral feeding as the best route for nutrition support, since it was better for the liver and kept the gut active [37, 42].

Some healthcare workers viewed vomiting of the tube, diarrhoea, and abdominal pain as possible negative consequences of nasogastric tube feeding [37, 42]. Some healthcare workers also mentioned aspiration as a possible risk of tube feeding [31, 37]. In one study, healthcare workers reported that clinicians’ fears of causing vomiting and abdominal pain in children could be a barrier to enteral feeding initiation [37]. A rare complication mentioned by one healthcare worker was an infection from the tape on the cheek [37].

A few healthcare workers described concerns about the discomfort for the child [37]. Some healthcare workers also seemed to be aware of parents’ concerns around the appearance of the tube, invasiveness of the procedure, discomfort for the child, or fears about returning to normal feeding [37, 42]. Some healthcare workers believed the insertion procedure could be experienced by parents as traumatic to watch [37, 42].

**Finding 13**: **Some healthcare workers reported that the introduction of nutritional tools**,** protocols or other interventions to improve practices could increase their knowledge and awareness of nutrition support**,** although a lack of time could be a barrier to using new tools. Some healthcare workers also emphasised the importance of including dietitians in care as this could be a valuable source of guidance**,** could improve nutritional practices**,** and could reduce healthcare workers’ workload (low confidence).**

In two studies, healthcare workers described how interventions to improve nutrition support had improved their knowledge and awareness of nutrition support [32, 34]. In one study, healthcare workers reported that implementing tools to calculate nutrition and a computerised protocol for nutrition support had improved their knowledge and attention to nutrition, making their practices more systematic and consistent [34]. However, in the same study, healthcare workers reported that lack of time was a barrier to using new tools [34].

Several healthcare workers described the significance of working together on nutrition across specialities, emphasising in particular the importance of including dietitians in care [31, 32, 37]. Healthcare workers described dietitians as a valuable resource for guidance on nutrition [37]. In a study about the introduction of a paediatric nutrition program led by a dietitian in a hospital in Malawi, a healthcare worker described dietetics as more concerned with involving parents and caregivers in nutrition support, which they saw as a benefit for the care when it needed to continue at home [32]. Other participants viewed the role of dietitians as one of educating the other professionals around nutrition support or connecting healthcare workers across different wards as the dietitian was one of the few people who worked in different wards across the hospital [32]. One healthcare worker also mentioned that having a dietitian could reduce workload by allowing other health professionals to focus on their main clinical roles [32]. Some healthcare workers also emphasised the need for government support to retain dietitians in hospitals [32].

**Finding 14: Some healthcare workers reported that a lack of resources could be a barrier to providing adequate nutritional support**,** for example when nutritional support was not consistently available or primarily made available to malnourished children**,** or where storage facilities for breast milk were lacking (moderate confidence).**

Healthcare workers in some studies described how a lack of various resources could present barriers to nutrition support [31, 32, 37]. In the study from Malawi, healthcare workers reported that prior to the implementation of the nutrition support intervention that was studied, nutrition support was only available in the nutrition support unit for malnourished children [32]. This meant if nutrition support was needed in other wards, for example before surgery, healthcare workers needed to ask for help from the nutritional rehabilitation unit (NRU), and this was not always given. In one study, healthcare workers reported that ensuring access to feeds could be an issue because of the cost of these [37]. In the study from Malawi, healthcare workers also reported that ensuring a consistent supply of feeds could be a challenge [32], though it was not clear if cost was the only issue that created challenges. The study from Ghana reported that a lack of storage facilities for breastmilk in ICUs meant that the babies would sometimes be fed with formula instead of expressed breastmilk, as intended [31].

### Review author reflexivity

At the beginning of the review process, none of the review authors had specific opinions on enteral feeding other than the awareness that early enteral feeding in critically ill children is a recommended practice. This was also true for the three co-authors with clinical experience in enteral feeding (CN, SMB, NMM). None of the authors had any personal experience with enteral nutrition as a recipient or as a caregiver to a child receiving enteral feeding. Moreover, except for CN, none of the review authors had carried out or were otherwise familiar with the research literature on enteral feeding before starting work on this review. However, based on our experience as health systems researchers, our preparatory reading of literature on the topic, as well as our initial experiences of review co-production with stakeholders, we expected to find a range of individual- and systems-level factors that may create barriers to the provision of enteral feeding.

On reflection, the views we held at the start of this review were largely the same at the end of the review process, although our understanding of the issues described above became more nuanced. Several findings were unexpected to us, for instance, the association made by parents between enteral feeding and the idea that their child is dying or very ill, or that children and parents might have concerns about the appearance of the tube. The review process has led us to recognise how important it is for healthcare workers to offer emotional support and communicate with parents about what healthcare workers may consider to be a relatively minor intervention. Overall, we have gained a better understanding of the potential discomfort of enteral feeding for parents as well as children, and this has led us to believe that this aspect of the intervention requires more attention in a clinical setting.

## Discussion

Our findings related to parents’ and children’s experiences and perceptions of enteral feeding indicated that parents and children have fears and concerns about discomfort, pain, and other complications of enteral feeding, while at the same time seeing benefits of the enteral feeding. While it may be likely that parents may be concerned about pain, discomfort and appearance of the feeding tube as suggested by these findings, it is also likely that parents will have more pressing concerns when their child is critically ill and in intensive care. Also, children may be less aware of the intervention due to the critical illness. Other systematic reviews have likewise reported parents’ experiences of stress and anxiety when taking care of newborns in the NICU [46] or when children were transferred from the PICU to the ward [47]. Apart from caring for a critically ill child, other sources of stress for parents could arise from strained family relations, the hospital environment, or anxiety over prognosis and outcomes [48].

Our findings suggested that receiving support, information, and participating in decision-making around enteral feeding was important to parents. Our findings also indicated that parents’ information needs were likely to differ at different stages of the child’s care and that parents might not remember information given at early and stressful stages of their child’s illness. This suggests that the timing of information is key. In addition, it is likely that information and involvement in decisions about enteral feeding are also often competing with other critical care interventions that are taking place at the same time. Similar findings were seen in qualitative evidence syntheses exploring parents’ experiences of NICUs and PICUs. These syntheses emphasised the importance of providing adequate and consistent information to parents and of involving them in decision-making [49–51], also describing how parents’ needs for information and involvement were likely to be shaped by factors such as parents’ individual preferences and the severity of the child’s condition [49, 51].

Our findings about healthcare worker’s experiences and perceptions of enteral feeding indicated that a lack of knowledge and skills to provide adequate nutritional support, variations in enteral feeding practices due to a lack of fixed rules and clear criteria, insufficient training, and a lack of resources could be barriers to optimal nutrition support. These findings also suggested that tools, protocols or other interventions to improve practices, including increased access to dietitians, could increase healthcare workers’ knowledge and awareness of nutrition support. In line with our findings, a mixed-methods review of clinicians’ knowledge, attitudes and beliefs about nutrition for critically ill adults in intensive care settings identified a lack of resources and nutrition protocols as barriers to adequate nutrition [19]. This review also found that factors such as the implementation of nutritional protocols, nutritional education, and the presence of supportive multidisciplinary teams could improve nutrition provision in critical care.

### Related evidence

As previously described, this QES was developed to inform a guideline development process by providing evidence to inform decision about possible acceptability, feasibility and equity implications of early versus delayed enteral feeding in critically ill children. In addition to the QES, project team members prepared a systematic review of randomised controlled trials (RCTs) to provide information about the relative effectiveness of early versus late enteral feeding, as well as an economic evaluation specifically relevant to the health system in Malawi. The economic evaluation, conducted as a scoping of economic evaluations, did not identify any existing evaluations appropriate for use in the Malawian context, so a new economic evaluation was therefore undertaken [52]. As was the case for the QES, the effectiveness systematic review identified few eligible studies, with only four RCTs being included in this review. All these RCTs were relatively small and their findings were assessed as having high or some concerns for overall risk of bias, leading the review team to assess the GRADE certainty of evidence as low to very low [53]. This limited evidence reduced confidence in findings on the effectiveness of early versus delayed enteral feeding for the prioritised clinical outcomes, although there was a signal of early enteral feeding being beneficial. The QES findings point to a range of issues that may influence the implementation of enteral feeding and thereby also influence its potential effectiveness and cost-effectiveness. Future trialists as well as implementers should therefore consider these factors.

### Limitations of the review

Our review found only 14 studies. This likely reflects a gap in the literature, but could also be due to some limitations in our search strategy. We searched databases that were likely to provide good access to qualitative studies. However, the inclusion of databases such as LILACs could have increased our chances of identifying studies from Latin American countries. Also, due to our limited capacity for translation, we only included studies in English. Including studies in languages other than English could potentially have added to the evidence base for this review. Finally, we did not search for grey literature.

### Implications for future research

The following implications for future research are based on where we found no or few studies, our methodological assessments of the included studies, and our GRADE-CERQual assessments of the review findings.

This review was carried out to provide evidence for a guideline development process that focused on the question of early enteral feeding compared to delayed enteral feeding in critically ill children. We found no studies from South-East Asia and few studies from non-anglophone countries. Few studies reported nutrition support practices in paediatric intensive care settings. Apart from the study by Moullet and colleagues [34], where healthcare workers reported that they generally introduced enteral feeding within 48 h, the studies we found did not mention the timing of enteral feeding. More qualitative research that explores early enteral feeding in intensive care settings could provide a better understanding of healthcare worker perceptions and practices around the timing of enteral feeding. We also did not find studies about parents’ and children’s experiences and perceptions of enteral feeding in critical care settings. More research about how parents and children experience enteral feeding in this setting is needed. Future studies that explore experiences of enteral feeding in acute care settings should aim to include children’s perspectives in order to provide a more in-depth understanding of children’s experiences as distinct from their parents’ experiences. In addition, future studies should focus on healthcare workers’ nutrition support practices in intensive care settings, and their practices related to communication and involvement of parents in decision-making practices.

In order to increase our confidence in the evidence, future qualitative studies should provide thicker description of informants’ views, practices, and experiences. Using participant or non-participant observation methods in addition to semi-structured interviews or focus groups could provide a fuller understanding of enteral feeding practices. In addition, the methodological quality of studies could be improved by sampling from a wider range of participants representing different socioeconomic status or cultural backgrounds; and detailed reporting of the sampling process. Studies should aim to show greater awareness of how researcher characteristics may influence the research process and study findings, i.e., reflexivity, and the studies should provide transparent reporting of these issues.

### Implications for practice

We have used our review findings to develop a set of questions intended for policymakers, hospital staff, managers, and others who are involved in the planning and implementation of enteral feeding for critically ill children. The questions are based on our review findings and address issues that the research suggests are important to parents, children and healthcare workers. These prompts are not intended to be recommendations but are phrased as questions to help implementers consider the implications of the review findings within their context. A printable version of the questions is available in Additional file 7.

#### How can you ensure that healthcare workers have access to nutritional training and support?

- Do hospital staff have regular training in feeding critically ill children? Does the timing of training take staff turnover into consideration? Does the training cover how to give information and support to parents, how to insert an enteral feeding tube and how to give feeds?
- Do staff have easy and timely access to dietitians, nutritionists or other appropriate personnel to consult as needed, including on children’s nutritional requirements and on appropriate feeds?
- Do staff have protocols for enteral feeding of critically ill children? Do they have tools to calculate nutritional needs? And have they received training in the use of these protocols and tools?

#### How can you ensure that healthcare facilities have reliable access to feeds and equipment?

- In some settings, specialised resources for enteral feeding are available only in departments that treat malnourished children. Do all hospital wards where enteral feeding is used have the necessary equipment to provide and manage the delivery of enteral feeding to children? Do all hospitals have the funding they need to cover the costs of enteral feeding for all wards that implement this, for instance, for commercial or specialised feeds and equipment?

#### How can you provide parents and children with adequate information and support at different stages of the enteral feeding process?

- Parents and children may find the tube insertion process uncomfortable or distressing, but enteral feeding sometimes takes place in situations where there is little time to give parents and children information and support. Consider how best to prepare children and parents for the insertion process, particularly in busy hospital wards. For instance, can staff explain what will happen and whether it will cause discomfort? Can parents be given the option of not being present when the tube is inserted if they feel it will be distressing?
- Hospital staff do not always have the knowledge and time to provide support or information and answer questions. In addition, parents may not remember information that is given when they are under stress. Can information be provided in a format that parents (and hospital staff) can access at any time point, for instance information leaflets, webpages or wall posters? Have you considered how to provide information to people who don’t speak the majority language or don’t read or write, for instance pictorial information?
- Parents may find it difficult to see their child being fed through a tube and may interpret it as an indication that the child is terminally ill or sicker than they actually are. How can staff provide parents with the reassurance and support they need?
- In addition to the support staff can provide, parents can be sources of support for each other. Can dedicated spaces be provided in the hospital where parents can meet each other? Can parents get help to establish or access peer support groups?
- Parents and older children are also likely to have questions after enteral feeding has started. Do parents have easy access to information that can answer questions including:
  - Why can’t my child eat?
  - Is the tube causing pain and discomfort?
  - How will my child’s skin react to the adhesive tape that may be used to fit the tube in place?
  - Will there be any side effects?
  - Will enteral feeding impact on my child’s growth?
  - How long will the tube stay in?
  - What can I do if my child experiences vomiting or nausea?

#### How can you ensure that parents are involved in the decision-making process?

- Have staff ensured that parents have given consent?
- Do staff have the skills and time they need to involve parents in the decisions? Have parents received enough information to participate meaningfully in decisions about, for example, type of enteral feeding, type of feed or how often the feed is given?
- Could shared decision-making aids be made available?

#### How can you support mothers who were breastfeeding when enteral feeding started?

- How can hospital staff help mothers develop or maintain bonding with their child when breastfeeding is not possible?
- How can staff help breastfeeding mothers maintain their breast milk supply? For instance, can they offer advice and support about how to express breastmilk or use a pump?
- Do mothers have easy access to a pump? In hospital wards where the milk is not used immediately, are there appropriate storage facilities for their breast milk?
- Can designated spaces be provided for mothers to have privacy when expressing milk? Mothers may be concerned if the breastmilk that they expressed was not used, or if their child is given other feeds than breastmilk. Do hospital staff take care to use the breastmilk or to explain why the milk was not used? If additional feeds are provided, do hospital staff explain to mothers why the breastmilk is not sufficient?

## Conclusion

Our review points to several factors that may influence the provision of enteral feeding for critically ill children, including parents’ access to information, support and decision-making; and healthcare workers’ access to nutritional knowledge, skills and resources. We have developed a set of questions that could help policymakers, hospital staff, managers, and others who are involved in the implementation of enteral feeding to address issues raised by our review findings when planning and implementing enteral feeding for critically ill children. More qualitative research on healthcare workers’ nutritional support practices in intensive care settings is needed to provide a better understanding of the barriers to adequate enteral feeding.

## Electronic supplementary material

Below is the link to the electronic supplementary material.

**Additional File 1:** Search Strategies.

**Additional file 2:** PROSPERO protocol.

**Additional file 3:** Differences between review and protocol.

**Additional file 4:** Characteristics of excluded studies.

**Additional file 5:** Characteristics of included studies.

**Additional file 6:** GRADE-CERQual Evidence Profile Table.

**Additional file 7:** Implications for practice.

**Additional file 8:** References to excluded studies.

**Additional file 9:** PRISMA reporting guidelines checklist.

## Data Availability

The data supporting the findings are available in the Interactive Summary of Qualitative Findings (iSoQ) table using this link: https://isoq.epistemonikos.org/preview/isoq/0ac750e3de027b57/652e6c3528370c15ee163726/public.

## Abbreviations

GRADE: Grading of Recommendations Assessment, Development and Evaluation
GRADE-CERQual: GRADE-Confidence in the Evidence from Reviews of Qualitative research
GI: Gastrointestinal
iSoQ: Interactive Summary of Qualitative Findings
NICU: Neonatal intensive care unit
NGT: Nasogastric tube
NRU: Nutritional rehabilitation unit
PICU: Paediatric intensive care unit
QES: Qualitative Evidence Synthesis
RCT: Randomised controlled trial
SoQF: Summary of Qualitative Findings
SAM: Severe acute malnutrition
