# Supplementary material for: Factors that influence the provision of enteral feeding for critically ill children: a qualitative evidence synthesis
Source: BMC Nutr. 2025 May 19;11:98. doi: 10.1186/s40795-025-01077-3 (PMC12087210; doi:10.1186/s40795-025-01077-3)
Supplement: Supplementary file 1 — Additional File 1: Search Strategies. [file 40795_2025_1077_MOESM1_ESM.docx]

## Additional file 1: Search strategies

**Search for existing QESes**

**Epistemonikos  (searched May 16, 2023 / May 19, 2023)**

|  | Enteral feeding | ((enteric* OR enteral* OR tube* OR gastric* OR support*) AND (nutrition* OR feeding*)) |
| --- | --- | --- |
|  | Children | child* OR infant* OR newborn* OR pediatr* OR paediatr* OR PICU* |
|  | critically ill when hospitalized OR intensive care | ((hospital* OR in-hospital* OR inpatient* OR in-patient*) AND ((critical* AND (ill OR illness) OR “high risk”)) OR ICU OR PICU* OR ((intensive OR critical*) AND care)) |

1 AND 2 AND 3 = 1 161 hits (see complete strategy below)

Limited to:

- Systematic review: 207
- Broad syntheses: 18
- Structured summary: 1

| 1 AND 2 AND 3 | (title:(((enteric* OR enteral* OR tube* OR gastric* OR support*) AND (nutrition* OR feeding*))) OR abstract:(((enteric* OR enteral* OR tube* OR gastric* OR support*) AND (nutrition* OR feeding*)))) AND (title:(child* OR infant* OR newborn* OR pediatr* OR paediatr* OR PICU*) OR abstract:(child* OR infant* OR newborn* OR pediatr* OR paediatr* OR PICU*)) AND (title:(((hospital* OR in-hospital* OR inpatient* OR in-patient*) AND ((critical* AND (ill OR illness) OR "high risk")) OR ICU OR PICU* OR ((intensive OR critical*) AND care))) OR abstract:(((hospital* OR in-hospital* OR inpatient* OR in-patient*) AND ((critical* AND (ill OR illness) OR "high risk")) OR ICU OR PICU* OR ((intensive OR critical*) AND care)))) |
| --- | --- |

**Search for primary studies**

**Ovid MEDLINE(R) and Epub Ahead of Print, In-Process, In-Data-Review & Other Non-Indexed Citations and Daily, 1946 to June 22, 2023 (searched June 26, 2023, updated July 3, 2024)**

| 1 | Enteral Nutrition/ | 21900 |
| --- | --- | --- |
| 2 | ((enteral or enteric or force or tube or gastric or intragastric or intestinal or intraintestinal or support) adj (nutrition* or feeding*)).tw. | 20328 |
| 3 | 1 or 2 | 31056 |
| 4 | exp Child/ | 2149098 |
| 5 | exp Infant/ | 1250315 |
| 6 | Minors/ | 2817 |
| 7 | exp Pediatrics/ | 63006 |
| 8 | exp Adolescent/ | 2214557 |
| 9 | (child* or infan* or newborn* or neonat* or kid or kids or boy or boys or girl or girls or underage* or under-age* or juvenil* or p?ediatric* or adolescen* or preteen* or midteen* or teen* or youth* or youngster*).tw. | 2794930 |
| 10 | (young* adj (person* or individual* or people* or male* or female*)).tw. | 83070 |
| 11 | or/4-10 | 4834319 |
| 12 | Hospitalization/ | 134747 |
| 13 | Inpatients/ | 29326 |
| 14 | (hospital* or inpatient*).tw. | 1666632 |
| 15 | or/12-14 | 1697316 |
| 16 | 11 and 15 | 433424 |
| 17 | Child, Hospitalized/ | 7350 |
| 18 | Adolescent, Hospitalized/ | 492 |
| 19 | or/16-18 | 435928 |
| 20 | 3 and 19 | 2489 |
| 21 | Interviews as Topic/ or Focus Groups/ or Narration/ or Qualitative research/ | 163116 |
| 22 | (("semi-structured" or semistructured or unstructured or informal or "in-depth" or indepth or "face-to-face" or structured or guide) adj3 (interview* or discussion* or questionnaire*)).tw,kf. | 176745 |
| 23 | (focus group* or interview or qualitative or ethnograph* or fieldwork or "field work" or key informant or mixed method*).tw,kf. | 523377 |
| 24 | or/21-23 | 631495 |
| 25 | 20 and 24 | 62 / 69 |
| 26 | (202306* or 202307* or 202308* or 202309* or 202310* or 202311* or 202312* or 2024*).dt,dp,ed,ep,yr. | 1902338 |
| 27 | 25 and 26 | 9* |

**CINAHL via Ebsco, 1981 to June 26, 2023 (searched June 26, 2023, updated July 3, 2024)**

| S1 | (MH "Enteral Nutrition") | 10801 |
| --- | --- | --- |
| S2 | TI ( ((enteral or enteric or force or tube or gastric or intragastric or intestinal or intraintestinal or support) N1 (nutrition* or feeding*)) ) OR AB ( ((enteral or enteric or force or tube or gastric or intragastric or intestinal or intraintestinal or support) N1 (nutrition* or feeding*)) ) | 15632 |
| S3 | 1 or 2 | 51401 |
| S4 | (MH "Child") OR (MH "Child, Preschool") OR (MH "Pediatrics+") OR (MH "Adolescence") | 952347 |
| S5 | (MH "Infant") OR (MH "Infant, Newborn+") | 284038 |
| S6 | TI ( (child* or infan* or newborn* or neonat* or p#ediatric* or kid or kids or minors or boy or boys or girl or girls or underage* or under-age* or juvenile* or adolescen* or preteen* or midteen* or teen* or youth* or youngster* or ((young N1 (person* or people* or individual* or male* or female*)) ) ) OR AB ( (child* or infan* or newborn* or neonat* or p#ediatric* or kid or kids or minors or boy or boys or girl or girls or underage* or under-age* or juvenile* or adolescen* or preteen* or midteen* or teen* or youth* or youngster* or ((young N1 (person* or people* or individual* or male* or female*)) ) | 1437398 |
| S7 | S4 OR S5 OR S6 | 1482697 |
| S8 | (MH "Hospitalization") | 46098 |
| S9 | (MH "Inpatients") | 86255 |
| S10 | TI ( hospital* or inpatient* ) OR AB ( hospital* or inpatient* ) | 585762 |
| S11 | S8 OR S9 OR S10 | 638159 |
| S12 | S7 AND S11 | 148292 |
| S13 | (MH "Child, Hospitalized") or (MH "Adolescent, Hospitalized") | 5329 |
| S14 | (MH "Infant, Hospitalized") | 807 |
| S15 | S12 OR S13 OR S14 | 149401 |
| S16 | S3 AND S15 | 1553 |
| S17 | TI interview OR AB interview | 226224 |
| S18 | (MH "Audiorecording") | 47126 |
| S19 | (MH "Qualitative Studies") | 142669 |
| S20 | TI qualitative stud* OR AB qualitative stud* | 82193 |
| S21 | S17 OR S18 OR S19 OR S20 | 331383 |
| S22 | S16 AND S21 - Limiters - Exclude MEDLINE records | 32 / 36 |
| S23 | EM 202306- | 274,481 |
| S24 | S22 AND S23 | 8* |

**Ovid Embase, 1974 to 2023 Week 25 (searched June 26, 2023, updated July 3, 2024)**

| 1 | Enteric Feeding/ | 40517 |
| --- | --- | --- |
| 2 | ((enteral or enteric or force or tube or gastric or intragastric or intestinal or intraintestinal or support) adj (nutrition* or feeding*)).tw. | 32722 |
| 3 | 1 or 2 | 51529 |
| 4 | Child/ or Preschool Child/ or School Child/ or Toddler/ or Childhood/ or exp Pediatrics/ | 2470293 |
| 5 | Infant/ or Baby/ or Newborn/ or Infancy/ | 1150717 |
| 6 | Adolescent/ or Adolescence/ | 1785529 |
| 7 | (child* or infan* or newborn* or neonat* or kid or kids or boy or boys or girl or girls or underage* or under-age* or juvenil* or p?ediatric* or adolescen* or preteen* or midteen* or teen* or youth* or youngster*).tw. | 3542121 |
| 8 | (young* adj (person* or individual* or people* or male* or female*)).tw. | 113553 |
| 9 | or/4-8 | 5046354 |
| 10 | Hospitalization/ | 528951 |
| 11 | Hospital Patient/ | 235566 |
| 12 | (hospital* or inpatient*).tw. | 2633483 |
| 13 | or/10-12 | 2788973 |
| 14 | 9 and 13 | 602875 |
| 15 | Hospitalized Child/ | 6118 |
| 16 | Hospitalized Infant/ | 1006 |
| 17 | Hospitalized Adolescent/ | 676 |
| 18 | or/14-17 | 603794 |
| 19 | 3 and 18 | 4322 |
| 20 | Qualitative Research/ | 116972 |
| 21 | (("semi-structured" or semistructured or unstructured or informal or "in-depth" or indepth or "face-to-face" or structured or guide) adj3 (interview* or discussion* or questionnaire*)).ti,ab. or (focus group* or qualitative or ethnograph* or fieldwork or "field work" or "key informant").tw,kw. | 589370 |
| 22 | 20 or 21 | 603320 |
| 23 | 19 and 22 | 75 |
| 24 | limit 23 to "remove medline records" | 38 / 42 |
| 25 | (202306* or 202307* or 202308* or 202309* or 202310* or 202311* or 202312* or 2024*).dc,dd,yr. | 2396578 |
| 26 | 24 and 25 | 4 |

**Scopus (Elsevier) (searched June 26, 2023, updated July 3, 2024)**

| 1 | TITLE-ABS-KEY ((enteral OR enteric OR force OR tube OR gastric OR intragastric OR intestinal OR intraintestinal OR support) PRE/1 (nutrition* OR feeding*)) | 49471 |
| --- | --- | --- |
| 2 | TITLE-ABS-KEY (child* or infan* or newborn* or neonat* or kid or kids or boy or boys or girl or girls or underage* or under-age* or juvenil* or pediatric* or adolescen* or preteen* or midteen* or teen* or youth* or youngster*) | 6415804 |
| 3 | TITLE-ABS-KEY (young* PRE/1 (person* or individual* or people* or male* or female*)) | 180346 |
| 4 | #2 OR #3 | 6505274 |
| 5 | TITLE-ABS-KEY (hospital* or inpatient*) | 2846704 |
| 6 | #4 AND #5 | 651251 |
| 7 | #1 AND #6 | 4822 |
| 8 | TITLE-ABS-KEY (qualitative OR interview* OR “thematic analysis” OR themes OR “mixed method” OR “mixed methods”) | 1980519 |
| 9 | #7 AND #8 | 147 |
|  | **Complete query – excluding MEDLINE records**  ( ( TITLE-ABS-KEY ( ( enteral OR enteric OR force OR tube OR gastric OR intragastric OR intestinal OR intraintestinal OR support ) PRE/1 ( nutrition* OR feeding* ) ) ) AND ( ( ( TITLE-ABS-KEY ( child* OR infan* OR newborn* OR neonat* OR kid OR kids OR boy OR boys OR girl OR girls OR underage* OR under-age* OR juvenil* OR pediatric* OR adolescen* OR preteen* OR midteen* OR teen* OR youth* OR youngster* ) ) OR ( TITLE-ABS-KEY ( young* PRE/1 ( person* OR individual* OR people* OR male* OR female* ) ) ) ) AND ( TITLE-ABS-KEY ( hospital* OR inpatient* ) ) ) ) AND ( TITLE-ABS-KEY ( qualitative OR interview* OR "thematic analysis" OR themes OR "mixed method" OR "mixed methods" ) ) AND NOT INDEX ( medline ) | 31 |
