# Supplementary material for: Factors that influence the provision of enteral feeding for critically ill children: a qualitative evidence synthesis
Source: BMC Nutr. 2025 May 19;11:98. doi: 10.1186/s40795-025-01077-3 (PMC12087210; doi:10.1186/s40795-025-01077-3)
Supplement: Supplementary file 2 — Additional file 2: PROSPERO protocol. [file 40795_2025_1077_MOESM2_ESM.pdf]

Factors that influence the provision of early enteral feeding for critically ill children in paediatric intensive care: a rapid qualitative evidence synthesis

To enable PROSPERO to focus on COVID-19 submissions, this registration record has undergone basic automated checks for eligibility and is published exactly as submitted. PROSPERO has never provided peer review, and usual checking by the PROSPERO team does not endorse content. Therefore, automatically published records should be treated as any other PROSPERO registration. Further detail is provided here.

Citation

Elisabeth Søiland, Susan Munabi-Babigumira, Claire Glenton, Lena Victoria Nordheim, Suzgika Lakudzala, Nyanyiwe Masinigi Mbeye, Idriss Ibrahim Kallon, Celeste Naude, Amanda Brand, Simon Lewin. Factors that influence the provision of early enteral feeding for critically ill children in paediatric intensive care: a rapid qualitative evidence synthesis. PROSPERO 2023 CRD42023432643 Available from:
https://www.crd.york.ac.uk/prospero/display_record.php?ID=CRD42023432643

Review question

To explore factors associated with the provision of early enteral nutrition for critically ill children in paediatric intensive care.

Searches

MEDLINE via Ovid, CINAHL via EBSCOhost, Embase via Ovid, Scopus via Elsevier.

We will search all databases from their inception to the search date.

Search strategy

https://www.crd.york.ac.uk/PROSPEROFILES/432643_STRATEGY_20230626.pdf

Types of study to be included

We will include primary studies that use qualitative study designs such as ethnography, phenomenology, case studies, grounded theory studies and qualitative process evaluations. We will include studies that use both qualitative methods for data collection (e.g., focus group discussions, individual interviews, observation, diaries, document analysis, open-ended survey questions) and qualitative methods for data analysis (e.g., thematic analysis, framework analysis, grounded theory). We will exclude studies that collect data using qualitative methods but do not analyse these data using qualitative analysis methods (e.g., open-ended survey questions where the response data are analysed using descriptive statistics only).

We will include both published and unpublished studies.

We will include mixed methods studies where it is possible to extract the data that were collected and analysed using qualitative methods.

We will include studies regardless of whether they were conducted alongside studies of the effectiveness of early enteral nutrition in critically ill children.

Condition or domain being studied

Nutritional support can be defined as the supply of energy (i.e., in the form of glucose, protein, or lipid) to provide calories (dietary energy) and substrate for metabolism (Joffe et al-, 2016). Evidence suggests that nutritional support is one of the key pillars to avoid undernourishment in critically ill children (Kratochvíl et al., 2022; Solana et al., 2023. Given that there may be a significant malnutrition rate (up to 25%) at the time of admission to the pediatric intensive care unit (PICU), nutritional support should be tailored to each child's specific needs (Kratochvíl et al., 2022).

Poor nutritional condition in the PICU is linked to post-discharge morbidity, higher mortality, and inpatient morbidity (Bunyani et al., 2015; Daniel et al., 2019; Kratochvíl et al., 2022; Tume et al., 2020). Paediatric patients who are malnourished at admission to PICU are at an increased risk of infection, death, prolonged mechanical ventilation and usage, as well as length of stay (Albadi & Bookari, 2022; Bagri et al., 2015). With few skilled persons available to give nutritional interventions, nutrition care for these children is highly limited in resource-constrained countries like Malawi.

Participants/population

This synthesis will focus on the views, attitudes, experiences and behaviours of children, caregivers, health care providers, and any other stakeholders potentially affected by a recommendation on the use of enteral feeding to critically ill children in a hospital setting, with a focus on early versus delayed introduction of enteral feeding, if possible.

By 'children' we mean persons between the age of 0 and 18. By 'caregivers', we mean anyone who is directly involved in caring for the child or making the decision of accepting the intervention on behalf of the child. By 'health care providers' we mean any cadre of healthcare workers involved in the provision of enteral nutrition for critically ill children, as well as any other staff involved in supporting the provision of enteral nutrition for critically ill children, e.g. administrative, managerial or supervisory staff.

We will exclude studies where the primary focus is on children with Severe Acute Malnutrition (SAM) or children with chronic health conditions (e.g., neurological impairment, genetic disorders) who require long-term enteral feeding

Intervention(s), exposure(s)

Enteral feeding is the provision of nutrition, most often in the form of a special liquid formula, using a tube feeding directly into the gastrointestinal tract bypassing the oesophagus. Most commonly, tube feeding is initiated using a nasogastric feeding tube (NGT), but nasoduodenal, nasojejunal, gastrostomy and jejunostomy tubes are also used to access the gastrointestinal tract.

Enteral nutrition has been shown to result in multiple benefits including fewer infections and better healing during ICU dependency, as well as overall, better short- and long- term clinical outcomes in paediatric critical care patients (Srinivasan et al., 2020). Timing is especially important and for enteral nutrition to be effective, it needs to be implemented early (within the first 48 hours after indication for enteral nutrition) as demonstrated in randomised control trials (Mart et al., 2023). Early enteral nutrition has been associated with better outcomes compared to delayed enteral nutrition, as it promotes and maintains gastrointestinal mucosal integrity including higher caloric intake and fewer complications. These findings suggest that early enteral nutrition could be a valuable intervention to improve outcomes in critically ill children requiring enteral nutrition in Malawi.

Comparator(s)/control

Not relevant

Context

By enteral feeding we mean the delivery of nutrition directly into the GI tract through a tube, e.g., a nasogastric tube, gastrostomy tube or jejunostomy tube. We will exclude studies that focus only on parenteral feeding, i.e., intravenous provision of nutrition.

We will include studies that focus on the provision of enteral feeding for critically ill children in any hospital setting, including general paediatric ward settings as well as acute hospital settings. We will exclude studies where the primary focus is on child enteral feeding in home or community settings.

Main outcome(s)

Not relevant

Measures of effect

Not relevant

Additional outcome(s)

Not relevant

Measures of effect

Not relevant

Data extraction (selection and coding)

We will develop a data extraction form for use when extracting the following descriptive information:

• First author of the study, year of publication, country of study, study setting (level of care).

• Participants' age, socioeconomic status, healthcare condition, cadre of health worker (e.g., nurse, doctor, midwife, or other cadre identified as the health provider), type of enteral feeding (e.g., nasogastric tube, gastrostomy tube, jejunostomy tube) and type of feed (e.g., commercial feed, hydrolysed feed, staple food, modified staple food)

• Any other information identified as important for subgroup analyses, such as the country setting (e.g., high- or low-/middle-income country setting)

• Information about study design and sources of funding

Secondly, we will extract all data relevant to the review objective. This includes data describing the views, experiences, and behaviour of children, parents, healthcare workers and others involved in the provision of enteral feeding of critically ill children. See further details under 'Data management, analysis and synthesis'.

One review author will extract the data, and another will cross-check the data to ensure that all relevant data has been extracted. Disagreements will be resolved by discussion or in consultation with a third review author.

Risk of bias (quality) assessment

At least two review authors will independently assess methodological limitations for each study using a quality assessment tool for qualitative studies used in previous Cochrane Reviews (Ames 2017; Ames 2019; Houghton 2020). Where any of the review authors are also authors of included studies, they will not be involved in assessing the study's methodological limitations. We will resolve disagreements by discussion or, when required, by involving a third review author.

We will assess methodological limitations according to the following domains.

• Are the settings and context described adequately?

• Is the sampling strategy described, and is this appropriate?

• Is the data collection strategy described and justified?

• Is the data analysis described, and is this appropriate?

• Are the claims made/findings supported by sufficient evidence?

• Is there evidence of reflexivity?

• Does the study demonstrate sensitivity to ethical concerns?

• Any other concerns?

We will report our assessments in a Methodological Limitations table, using a 'yes/no/uncertain' rating and with explanations of any concerns we have. We will use these assessments to support our GRADE-CERQual (Confidence in the Evidence from Reviews of Qualitative research) assessment of our confidence in the review findings (Lewin 2018).

Strategy for data synthesis

We will use a thematic synthesis method as our analytical approach (Thomas & Harden, 2008).We will select the article that most closely answers the review objectives and create a data extraction sheet based on these codes. Two review authors will then code the data extracted from this article. These authors will then code the subsequent articles using the data extraction sheet, adding new codes from subsequent articles as they emerge. Data extraction will be verified by other review authors. Review findings will then be synthesised from the data that has been given the same codes across the studies. Findings will be shared with co-authors to review. Finally, we will re-read the included studies to check that we have extracted all data relevant to the findings.

Analysis of subgroups or subsets

We will consider the need for subgroups analyses during the data extraction phase.

Contact details for further information

Elisabeth Søiland

Elisabeth.Soiland@hvl.no

Organisational affiliation of the review

Western Norway University of Applied Sciences

www.hvl.no

Review team members and their organisational affiliations

Elisabeth Søiland. Western Norway University of Applied Sciences

Susan Munabi-Babigumira. Western Norway University of Applied Sciences

Claire Glenton. Western Norway University of Applied Sciences

Lena Victoria Nordheim. Western Norway University of Applied Sciences

Suzgika Lakudzala. Kamuzu University of Health Sciences

Nyanyiwe Masinigi Mbeye. Kamuzu University of Health Sciences

Idriss Ibrahim Kallon. Stellenbosch University

Celeste Naude. Stellenbosch University

Amanda Brand. Stellenbosch University

Simon Lewin. Norwegian University of Science and Technology

Collaborators

Rose Chipojola. Kamuzu University

Gertrude Kunje. Kamuzu University

Talitha Mpando. Kamuzu University

Marianne Visser. Stellenbosch University

Sarah Gordon. Stellenbosch University

Dachi Arikpo. Cochrane Nigeria

Moriam Chibuzor. Cochrane Nigeria

Pamela Vorster. Stellenbosch University

Elodie Besnier. Norwegian University of Science and Technology

Type and method of review

Synthesis of qualitative studies, Systematic review

Anticipated or actual start date

14 April 2023

Anticipated completion date

22 December 2023

Funding sources/sponsors

This review is part of the GELA project which is funded by the European and Developing Countries Clinical Trials Partnership. This project is part of the EDCTP2 programme supported by the European Union

Grant number(s)

State the funder, grant or award number and the date of award

The European and Developing Countries Clinical Trials Partnership. Grant number RIA2020S-3303-GELA, awarded 1 April 2022

Conflicts of interest

Language

English

Country

Malawi, Nigeria, Norway, South Africa

Published protocol

https://www.crd.york.ac.uk/PROSPEROFILES/432643_PROTOCOL_20230626.pdf

Stage of review

Review Ongoing

Subject index terms status

Subject indexing assigned by CRD

Subject index terms

Child; Critical Care; Critical Illness; Enteral Nutrition; Humans; Intensive Care Units, Pediatric

Date of registration in PROSPERO

06 July 2023

Date of first submission

26 June 2023

Stage of review at time of this submission

The review has not started

**Stage**	**Started**	**Completed**
Preliminary searches	No	No
Piloting of the study selection process	No	No
Formal screening of search results against eligibility criteria	No	No
Data extraction	No	No
Risk of bias (quality) assessment	No	No
Data analysis	No	No

The record owner confirms that the information they have supplied for this submission is accurate and complete and they understand that deliberate provision of inaccurate information or omission of data may be construed as scientific misconduct.

The record owner confirms that they will update the status of the review when it is completed and will add publication details in due course.

Versions

06 July 2023

06 July 2023
