# Supplementary material for: Factors that influence the provision of enteral feeding for critically ill children: a qualitative evidence synthesis
Source: BMC Nutr. 2025 May 19;11:98. doi: 10.1186/s40795-025-01077-3 (PMC12087210; doi:10.1186/s40795-025-01077-3)
Supplement: Supplementary file 3 — Additional file 3: Differences between review and protocol. [file 40795_2025_1077_MOESM3_ESM.docx]

**Additional file 3: Differences between protocol and review**

The protocol for this review was registered to the international prospective register of systematic reviews, PROSPERO ([CRD42023432643](https://www.crd.york.ac.uk/prospero/display_record.php?ID=CRD42023432643)).

We decided to remove the reference to chronic health conditions in the description of the exclusion criteria, instead, using only the home setting as the exclusion criteria. We did this because we saw that although children with chronic health conditions were more likely to receive long-term feeding in the home, which was out of the scope of our research question, some of these children may initially have received a recommendation for enteral feeding during a critical phase of illness when they were in hospital. These experiences would then have been relevant for our research question, so we decided to not exclude children with chronic health conditions as a population.

However, after full-text screening we decided to add the patient population of children with anorexia to the exclusion criteria. We did this because we considered the experiences of children who were malnourished prior to hospital admission and received tube feeding against their will would be too different from the experiences from children who did not have any difficulties eating or malnourishment prior to hospital admission.

We also decided to remove reference to long-term enteral feeding in the protocol exclusion criteria. We did this because we found that in the studies it could be difficult to distinguish between short- and long-term feeding at the time of initiation, as duration may not have been decided at the start of enteral feeding. Instead, we decided to focus on the whether the enteral feeding was happening in hospital or at home when deciding what studies to include.

We also did not use machine learning in the screening and study selection process, as the number of studies we found was low enough to efficiently screen without needing machine learning functions.

Due to the relatively low number of included studies, we decided not to select a sample of studies. Instead, we decided to extract data from all included studies.
