# Supplementary material for: Factors that influence the provision of enteral feeding for critically ill children: a qualitative evidence synthesis
Source: BMC Nutr. 2025 May 19;11:98. doi: 10.1186/s40795-025-01077-3 (PMC12087210; doi:10.1186/s40795-025-01077-3)
Supplement: Supplementary file 4 — Additional file 4: Characteristics of excluded studies. [file 40795_2025_1077_MOESM4_ESM.docx]

**Additional file 4: Characteristics of excluded studies**

| Study | Reason for exclusion |
| --- | --- |
| Abeasi 2020 | Wrong intervention |
| Abuidhail 2017 | Wrong study aim |
| Beck 2017 | Wrong study aim |
| Bicakli 2016 | Duplicate |
| Brotherson 1995 | Wrong setting |
| Brotherton 2009 | Wrong setting |
| Brotherton 2007a | Wrong setting |
| Brotherton 2007b | Wrong setting |
| Bucher 2018 | Wrong intervention |
| Bunyani 2015 | Wrong study design |
| Chaplen 1997 | Wrong study design |
| Cipolla 2022 | Wrong setting |
| Craig 2013 | Wrong study design |
| Craig 2003 | Wrong setting |
| Dadich 2023 | Wrong setting |
| Depianti 2023 | No relevant data |
| Edwards 2016 | Wrong intervention |
| Fleming 2015 | Wrong intervention |
| Guerriere 1998 | Wrong study design |
| Guerriere 2003 | Wrong setting |
| Gunes 2023 | Wrong study design |
| Halse 2005 | Wrong patient population |
| Herrington 2001 | Wrong setting |
| Hopwood 2020 | Wrong setting |
| Hopwood 2022 | Wrong patient population |
| Huhmann 2008 | Wrong study design |
| Keller 2014 | Wrong patient population |
| Kodua 2020 | Wrong intervention |
| Leong 2014 | Wrong study design |
| Lindberg 2014 | Wrong intervention |
| Lively 2021 | Wrong study aim |
| Mahant 2018 | Wrong study design |
| Mahant 2011 | Wrong study design |
| Michaelis 1992 | Wrong study design |
| Morris 2014 | Wrong patient population |
| Morrow 2008 | Wrong setting |
| Murphy 2023 | Wrong intervention |
| Namnabati 2017 | Wrong setting |
| Neiderman 2001 | Wrong patient population |
| Nelson 2015 | Wrong setting |
| Pinch 1990 | Wrong study aim |
| Poh 2024 | Wrong setting |
| Radford 1997 | Wrong setting |
| Ridley 2020 | Wrong study design |
| Rouse 2002 | Wrong setting |
| Sleigh 2005 | Wrong setting |
| Soscia 2021 | Wrong setting |
| Spalding 1998 | Wrong setting |
| Sullivan 1992 | Wrong study design |
| Swanson 2012 | Wrong intervention |
| Syrmis 2019 | Wrong study aim |
| Thomas 2024 | No relevant data |
| Townsley 1999 | Wrong setting |
| Wigert 2006 | Wrong study aim |
| Wilken 2012 | Wrong study design |
