# Supplementary material for: Factors that influence the provision of enteral feeding for critically ill children: a qualitative evidence synthesis
Source: BMC Nutr. 2025 May 19;11:98. doi: 10.1186/s40795-025-01077-3 (PMC12087210; doi:10.1186/s40795-025-01077-3)
Supplement: Supplementary file 5 — Additional file 5: Characteristics of included studies. [file 40795_2025_1077_MOESM5_ESM.docx]

**Additional file 5: Characteristics of included studies**

| **Study** | **Country** | **Country income level** | **Aim of the study** | **Study setting** | **Route(s) of enteral feeding studied** | **Content of feeds provided** | **Early or late initiation** | **Who initiated and provided the enteral feeding?** | **Type and number of informants** | **Age of children in study** | **Type of health care condition (children)** |
| --- | --- | --- | --- | --- | --- | --- | --- | --- | --- | --- | --- |
| Abukari 2021 | Ghana | Lower middle-income country | "The aim of this study was to explore and describe the feeding criteria of critically ill children at the neonatal and paediatric intensive care units." | NICU /PICU | NGT and parenteral | Informants referred to the use of formula, expressed breast milk, and mix-feeding | Not described (could be early) | Informants were selected because they directly handled the feeding process, but one informant mentions that “we do collaborative care where we involve other specialists like the nutritionists and dietitians, eye specialists, physiotherapists and other team members to share ideas and skills on how to go about the feeding." | 42 paediatric nurses, paediatricians, neonatal nurses, registered nurses, and midwives working at the neonatal and paediatric intensive care units for at least 2 years. | 0-12 years | Critically ill children in intensive care units |
| Banhara 2020 | Brazil | Upper middle-income country | "To understand the psychosocial repercussions experienced by caregiving parents resulting from the care of children with cleft lip, dysphagia and feeding tube." | A public, tertiary, referral hospital for craniofacial anomalies and related syndromes | NGT only | Some data describes breast milk fed through the tube | Not described | Not clear who initiated and provided the feeds | Mothers (7), ages 20 to 35. Married, belonging to low social class, and incomplete high school. | Not described | Cleft lip with dysphagia |
| Bicakli 2019 | Turkey | Upper middle-income country | "…to reveal children’s and their mothers’ views and experiences related to EN..." | Clinic inpatient service of a university paediatric oncology department | NGT only | Breastmilk and formula are mentioned | Not described | HCW-initiated and parent-provided | Children (n=3) and mothers (n=14) | 11mos-18yrs | Diagnosed with paediatric cancer |
| Cohen 2017 | Australia | High-income country | "To compare and contrast views among parents, patients and health-care workers on the positive and negative aspects of ETF, the ways in which information was provided on ETF and how the decision-making process was conducted for the initiation of ETF" | Hospital / cancer ward | 22 of the 30 children in the study have received nasogastric tube feeding. Unclear what other routes the other children have received, possibly gastrostomy. | Generally not described. One participant (mother for 5 year old child) described switching from ordinary milk to pre-digested feeds' . | Not described | Not described | Mothers (18), fathers (2), children (10) healthcare workers (18) | Under 18 (mean: 9,5 years, SD 5 years) | Paediatric cancer |
| Daniel 2019 | Malawi | Low-income country | "We aimed to further understand the phases before the implementation of nutrition support, during the introduction of the program, and the future of the program by undertaking qualitative interviews with health professionals at QECH who worked alongside the dietitian." | Hospital | Via NGT and gastrostomy, as well as nutrition support (therapeutic feeds) given orally | Infant formula and breastmilk (fortified in some cases) and therapeutic feeds (F-75, F-100, and ready-to-use-therapeutic foods; given to acutely malnourished children) | Not described (could be early) | Dietitians | Nurses (n=5) and physicians (n=11) | Not described | Paediatric patients triaged at the paediatric emergency department, admitted for chronic conditions, trauma including burns, andacute illnesses and infections. |
| Ellerton 1985 | Canada | High-income country | "to explore the way in which chronically ill hospitalized children expressed their concerns about intrusion during play interviews" (p. 168) | Children's ward | NGT only | Not described | Not described | Not described | Children (10) | 2-6 years (6 under 4 years, 4 over 4 years) | Chronic illness |
| Ferguson 2007 | Australia | High-income country | The aim of this study was to use  a qualitative research paradigm to  describe the parental experience of  caring for a child who received some  or all of their nutrition via a feeding  tube | Neonatal unit at major paediatric hospital | NGT only | Not described. | Not described (could be early) | Not specified | Families of six infants, between the age of four and ten months were selected | 4- 10 Months | Complex medical conditions |
| Lively 2023 | Australia | High-income country | "this study investigates parents' thoughts, experiences and reflections as they made the decision to, and then participated in, an intensive tube weaning programme." | Inpatient feeding therapy program | Naso-gastric tube feeding | Not described. | Not described. | Not described. | Fourteen families: mothers (n=9); fathers (n=2); mother and father dyads (n=3) | Not described. | Complex medical conditions including premature birth |
| Madiba 2021 | South Africa | Upper-middle income country | To explore and describe the experiences of mothers of preterm infants regarding initiation and expressing breast milk, nasogastric tube feeding practices, and transition to breastfeeding during the infants’ stay in the NICU and KMC unit of a South African academic hospital. | Kangaroo mother care unit of a tertiary hospital | NGT | Breastmilk | Not described | Not described for initial NICU feeding; mothers and HCW in the KMC unit | Mothers of preterm infants (n~35-40) "Data saturation was reached after 5 focus groups had been conducted with about 7 to 8 mothers in each group." | Newborn | Premature birth or illness |
| Montgomery 2013 | USA | High income country | To describe the perceptions of paediatric oncology patients and parents regarding the use of EN and PN interventions and identify variables influencing those perceptions. | Outpatient oncology service at a large Midwestern children’s hospital- approx. 200 children are seen in outpatient clinic every month | Enteral and parenteral feeding | Not described. | Not described. | Not described. | Children (15) and parents of children (32) receiving cancer treatment | 6 to over 18 years. 2 of the 15 children were between 18-21 years of age | Majority had haematological malignancy, rest had solid tumour. |
| Moullet 2020 | Switzerland | High-income country | "We aimed to explore the perceptions of PICU physicians about their management of EN and the implementation of a nutrition protocol and computerised system." | PICU | Mostly post pyloric or gastric, continuous | not described | Variable / early. "...they introduced EN within 6-48 h of PICU admission and recommended EN, with a continuous feeding, at post pyloric or gastric sites in older patients who tolerated this nutrition" | "Nutritional support was prescribed by physicians in the PICU, who may call a dietitian from the nutrition service in case of questions." | 40 interviews conducted with 33 physicians in a staged approach (stage 1=6 junior physicians, 5 fellows; stage 2=12 junior physicians, 12 Fellows, 5 senior physicians) | Not described. | Critically ill children |
| Mörelius 2020 | Sweden | High-income country | "To describe mother's experiences of feeding their extremely preterm infant." | Home, following discharge from NICU, as well as NICU | NGT inly (continuous or intermittent) | Breastmilk predominantly, some infant formula | Not described. | Not explicitly described, but it is stated that nurses working in the NICU provided breastfeeding support. Mothers | Mothers (10) | Extremely preterm infants born between gestational age 24 and 27 (mean: 26) weeks. | Extremely preterm (< 28 weeks gestational age) |
| Remijn 2022 | Netherlands | High-income country | (1) to evaluate parents’ experiences and expectations about tube feeding, and (2)to explore healthcare professionals’ suggestions for improving the management of tube feeding in children | Home following discharge from hospital | Nasogastric or gastrostomy | Not described. | Not described. | Not very clear, but is mentioned that Parents provided the feed at home. Nurses/Health care professions could come home and change the feeding tube | 17 Parents whose children had difficulty in feeding. 5 Health Care Professionals (dietitians, paediatricians, speech therapists, psychologists and nurse) | 0-10 years | Multiple diagnoses: feeding difficulties among children with Heart diseases Gastroesophageal reflux/gastric problems, Prematurity, Neurodevelopmental disorder, syndrome, no specific diagnosis |
| Willams-Hooker 2015 | USA | High-income country | To determine the preferences of the caregivers of paediatric patients undergoing Hematopoietic Stem Cell Transplant and those of the HC providers working on the HSCT unit and the reservations they had regarding both types of NS.' | HSCT unit at St. Jude hospital, provides care for paediatric patients with catastrophic disease. 19 inpatient rooms, with at least 90% of patients receiving NG feeding during their stay. | Enteral feeding compared to parenteral feeding | Not described | Not described. | Not described. | Total participants (caregivers and HC providers) included 17 men and 37 women, aged 22–59 years. | Not described. The study did not collect demo-graphic details of the patients. | Paediatric cancer |
| **Abbreviations**  EN: Enteral nutrition  ETF: Enteral tube feeding  HC providers: Healthcare providers  HCW: Healthcare workers  HSCT: Hematopoietic stem cell transplantation  KMC: Kangaroo Mother Care  NGT: Nasogastric feeding tube  NS: Nutrition support  NICU: Neonatal intensive care unit  PICU: Paediatric intensive care unit  PN: Parenteral nutrition | | | | | | | | | | | |
