# Supplementary material for: Factors that influence the provision of enteral feeding for critically ill children: a qualitative evidence synthesis
Source: BMC Nutr. 2025 May 19;11:98. doi: 10.1186/s40795-025-01077-3 (PMC12087210; doi:10.1186/s40795-025-01077-3)
Supplement: Supplementary file 6 — Additional file 6: GRADE-CERQual Evidence Profile Table. [file 40795_2025_1077_MOESM6_ESM.docx]

### **Additional file 6. GRADE CERQual Evidence Profile Table**

| **#** | **Summarised review finding** | **Methodological limitations** | **Coherence** | **Adequacy** | **Relevance** | **GRADE-CERQual assessment of confidence** | **References** |
| --- | --- | --- | --- | --- | --- | --- | --- |
| 1 | Parents and older or adolescent children described being worried about discomfort, pain, and other complications of tube feeding before enteral feeding was initiated. Their anxiety appeared to be related to a lack of knowledge and experience of enteral feeding. | No/Very minor concerns | No/Very minor concerns | Minor concerns  **Explanation:** Minor concerns regarding adequacy because the data is thin. | Minor concerns  **Explanation:** Moderate concerns regarding relevance. The underlying data is from non-intensive care settings and we are uncertain whether this finding is also relevant for parents whose children are in intensive care. For instance, these parents may have more pressing concerns because the child is acutely ill or may not be aware that their children are being tube-fed. We are also uncertain whether this finding is relevant for children in intensive care, as they may be unconscious and unaware of the initiation of enteral feeding. | Moderate confidence  **Explanation:** Minor concerns regarding adequacy because of thin data. Moderate concerns regarding relevance because of indirect data (from non-intensive care settings). No or very minor concerns regarding methodological limitations and coherence. | Banhara et al. 2020; Bicakli et al. 2019; Cohen et al. 2017; Daniel et al. 2019; Ferguson & Paul 2007; Madiba & Sengane 2021; Montgomery et al. 2013; Williams-Hooker et al. 2015; |
| 2 | Where parents were present during nasogastric tube insertion, some found the procedure difficult to watch because of the discomfort they observed their child experiencing. Other parents focused on how the discomfort did not last long and on the fact that the child recovered quickly. | No/Very minor concerns | No/Very minor concerns | Moderate concerns  **Explanation:** Moderate concerns regarding adequacy because few studies with thin data support the finding. | Minor concerns  **Explanation:** Minor concerns regarding relevance. The underlying data is from non-intensive care settings and we are uncertain whether this finding is also relevant for parents whose children are in intensive care. For instance, these parents may have more pressing concerns because the child is ill. | Moderate confidence  **Explanation:** Moderate concerns regarding adequacy because of thin data from few studies. Minor concerns regarding relevance because of indirect data (from non-intensive care settings). No or very minor concerns regarding methodological limitations and coherence. | Bicakli et al. 2019; Cohen et al. 2017; Ferguson & Paul 2007; Madiba & Sengane 2021; |
| 3 | Some parents felt more positive about nasogastric tube feeding after the tube had been inserted. Some reported that their children seemed to get used to the tube easily, while others reported seeing benefits of enteral feeding such as weight gain, better nutrition, easier administration of medication, and a less stressful feeding situation. Some older children and adolescents appeared to agree, although they expressed more ambivalent or negative feelings about the tube. Other parents and older children also described specific challenges during tube feeding such as vomiting, diarrhoea or other kinds of discomfort, and parents described how these challenges caused new concerns. | No/Very minor concerns | No/Very minor concerns | Moderate concerns  **Explanation:** Moderate concerns regarding adequacy because each part of the finding is supported by few studies and thin data. | No/Very minor concerns | Moderate confidence  **Explanation:** Moderate concerns regarding adequacy becuase of thin data from few studies. No or very minor concerns regarding methodological limitations, coherence, and relevance. | Banhara et al. 2020; Bicakli et al. 2019; Cohen et al. 2017; Ellerton et al. 1985; Ferguson & Paul 2007; Lively et al. 2023; Madiba & Sengane 2021; Montgomery et al. 2013; Mӧrelius et al. 2020; Remijn et al. 2022; |
| 4 | Before the nasogastric tube was inserted, some children and parents expressed concern about what the tube would look like. After insertion, some parents described getting used to seeing their child with the tube, although some older children and adolescents continued to feel self-conscious about their appearance. | Minor concerns  **Explanation:** Minor concerns regarding methodological limitations because some of the studies have unclear sampling strategies and unclear reporting of the samples, so it is unclear whether different groups are represented. Also, the lack of reflexivity around researcher roles could be a concern here since this finding is slightly more positive, suggesting that informants could have been influenced by wanting to maintain a positive relationship with researchers who were also healthcare workers. | No/Very minor concerns | Serious concerns  **Explanation:** Serious concerns regarding adequacy because the finding is supported by very few studies that all have very thin data. | Minor concerns  **Explanation:** Minor concerns regarding relevance. The underlying data is all from non-intensive care setting and we are uncertain whether this findings is also relevant for parents whose children are in intensive care. For instance, these parents may have more pressing concerns because the child is acutely ill. | Very low confidence  **Explanation:** Serious concerns regarding adequacy because of limited and very thin data (3 studies). Minor concerns regarding methodological limitations because of inadequate sampling and reporting of sampling and reflexivity. Moderate concerns regarding relevance because of indirectness (data from non-intensive care settings). No or very minor concerns regarding coherence. | Bicakli et al. 2019; Cohen et al. 2017; Ferguson & Paul 2007; |
| 5 | Mothers of preterm and critically ill newborns described tube feeding as emotionally challenging. These mothers said they felt frustrated when they could not breastfeed, and they reported feeling that tube feeding disrupted bonding and attachment. Some mothers felt excluded and powerless when healthcare workers took over the care of their infants, but they also reported needing support from healthcare workers as well as partners and others. Many mothers described transitioning from tube feeding to breastfeeding as a positive experience, but some mothers were concerned about weight loss due to the infant’s poor suckling abilities. | No/Very minor concerns | No/Very minor concerns | Serious concerns  **Explanation:** Serious concerns regarding adequacy because the finding is supported by few studies with thin data. | Minor concerns  **Explanation:** Minor concerns regarding relevance because mothers may not necessarily perceive breastfeeding as equally important for bonding in all settings. Also, data from one of the studies was only partially relevant to the finding because it was about breastfeeding in older children. | Low confidence  **Explanation:** Serious concerns regarding adequacy because of thin data from few studies. Minor concerns regarding relevance because the finding may not be relevant in all settings. No or very minor concerns regarding methodological limitations and coherence. | Banhara et al. 2020; Cohen et al. 2017; Madiba & Sengane 2021; Mӧrelius et al. 2020; |
| 6 | Healthcare workers and mothers of preterm and critically ill newborns described how expressing breast milk and sustaining milk supply while the infant was tube feeding was difficult. Although mothers were highly motivated to provide breastmilk for their infant, they said they needed support from healthcare workers, partners and other parents to succeed. | No/Very minor concerns | No/Very minor concerns | Moderate concerns  **Explanation:** Moderate concerns regarding adequacy because the finding is based on few studies. | No/Very minor concerns | Moderate confidence  **Explanation:** Moderate concerns regarding adequacy because of few studies. No or very minor concerns regarding methodological limitations, coherence, and relevance. | Abukari & Acheampong 2021; Madiba & Sengane 2021; Mӧrelius et al. 2020; |
| 7 | While some parents described a sense of participation in the decision-making process on whether to initiate enteral feeding for their child, others described how they were not involved in decisions related to this. Some healthcare workers agreed that parents should be more involved. | No/Very minor concerns | No/Very minor concerns | Serious concerns  **Explanation:** Serious concerns regarding adequacy because the finding is based on few studies, and even fewer studies support each part of the finding. The data is also very thin. | No/Very minor concerns | Low confidence  **Explanation:** Serious concerns regarding adequacy because of few studies and very thin data. No or very minor concerns regarding methodological limitations, coherence, and relevance. | Cohen et al. 2017; Daniel et al. 2019; Ferguson & Paul 2007; Mӧrelius et al. 2020; Remijn et al. 2022; |
| 8 | Some parents described how the hospital environment offered opportunities to exchange support with parents in similar situations and could help normalise the process. Other parents felt that the lack of support from staff made the hospital a difficult environment. | No/Very minor concerns | No/Very minor concerns | Serious concerns  **Explanation:** Serious concerns regarding adequacy because few studies and very thin data support the finding. | No/Very minor concerns | Low confidence  **Explanation:** Serious concerns regarding adequacy because of very thin data from few studies. No or very minor concerns regarding methodological limitations, coherence, adequacy, and relevance. | Ferguson & Paul 2007; Mӧrelius et al. 2020; Remijn et al. 2022; |
| 9 | Some parents reported receiving information about enteral feeding from hospital staff, while other parents said the information they had been given was not enough. Parents’ perception of the adequacy of the information they receive could be related to the way the information was conveyed. A few parents sought information from other sources than healthcare workers, including from other families. | No/Very minor concerns | No/Very minor concerns | Serious concerns  **Explanation:** Serious concerns regarding adequacy because the finding is supported by few studies and thin data. | No/Very minor concerns | Low confidence  **Explanation:** Serious concerns regarding adequacy because of thin data and few studies. No or very minor concerns regarding methodological limitations, coherence, adequacy, and relevance. | Cohen et al. 2017; Ferguson & Paul 2007; Mӧrelius et al. 2020; Remijn et al. 2022; |
| 10 | Some parents said they would have wanted some types of information at earlier stages, for example about duration and consequences of enteral feeding. Healthcare workers agreed that parents needed adequate information at each stage of enteral feeding, but both parents and healthcare workers reported that parents might not remember information given at early and stressful stages of their child’s illness. | No/Very minor concerns | No/Very minor concerns | Serious concerns  **Explanation:** Serious concerns regarding adequacy because the finding is supported by few studies with thin data. | No/Very minor concerns | Low confidence  **Explanation:** Serious concerns regarding adequacy because of thin data from few studies. No or very minor concerns regarding methodological limitations, coherence, and relevance. | Cohen et al. 2017; Ferguson & Paul 2007; Remijn et al. 2022; |
| 11 | Some healthcare workers reported that they lacked knowledge and skills to provide adequate enteral feeding for critically ill children. They described variations in practice due to a lack of clear criteria for initiation. Some healthcare workers who had received some training in nutrition support suggested that the training was insufficient. | No/Very minor concerns | No/Very minor concerns | Serious concerns  **Explanation:** Serious concerns regarding adequacy because the finding is supported by only few studies and thin data. | No/Very minor concerns | Low confidence  **Explanation:** Serious concerns regarding adequacy because of thin data from few studies. No or very minor concerns regarding methodological limitations, coherence, and relevance | Abukari & Acheampong 2021; Cohen et al. 2017; Moullet et al. 2020; |
| 12 | Healthcare workers described the main benefits of enteral feeding as ensuring adequate nutrition and weight gain or maintenance. Some healthcare workers also suggested that enteral feeding could lead to better health outcomes and reduced risk of death; shorter hospital stays and reduced risks of readmission; and improved tolerance among children of clinical interventions. Healthcare workers also believed that enteral feeding not only made it easier to feed the child but also to give medication. Healthcare workers described potential negative consequences of tube feeding as including vomiting, diarrhoea, abdominal pain, and the risk of aspiration. Some healthcare workers were concerned about the discomfort for the child as well as parents’ concerns about these negative consequences. | No/Very minor concerns | No/Very minor concerns | Serious concerns  **Explanation:** Serious concerns regarding adequacy because the finding is supported by few studies and thin data. | No/Very minor concerns | Low confidence  **Explanation:**  Serious concerns regarding adequacy because of thin data from few studies. No or very minor concerns regarding methodological limitations, coherence, and relevance. | Abukari & Acheampong 2021; Cohen et al. 2017; Daniel et al. 2019; Williams-Hooker et al. 2015; |
| 13 | Some healthcare workers reported that the introduction of nutritional tools, protocols or other interventions to improve practices could increase their knowledge and awareness of nutrition support, although a lack of time could be a barrier to using new tools. Some healthcare workers also emphasised the importance of including dietitians in care as this could be a valuable source of guidance, could improve nutritional practices, and could reduce their workload. | Minor concerns  **Explanation:** Minor concerns regarding methodological limitations because the lack of reflexivity by the study authors may have influenced the design and conduct of the studies. | No/Very minor concerns | Serious concerns  **Explanation:** Serious concerns regarding adequacy because the finding is supported by few studies, some of which offer very thin data. | No/Very minor concerns | Low confidence  **Explanation:** Serious concerns regarding adequacy because of thin data from few studies. Minor concerns regarding methodological limitations because study authors' lack of reflexivity may have influenced the design and conduct of the studies. No or very minor concerns regarding coherence and relevance. | Abukari & Acheampong 2021; Cohen et al. 2017; Daniel et al. 2019; Moullet et al. 2020; |
| 14 | Some healthcare workers reported that a lack of resources could be a barrier to providing adequate nutritional support, for example when nutritional support was not consistently available or primarily made available to malnourished children, or where storage facilities for breast milk were lacking. | No/Very minor concerns | No/Very minor concerns | Moderate concerns  **Explanation:** Moderate concerns regarding adequacy. Although the finding is based on few studies with thin data, we consider the data sufficient to support this finding. | No/Very minor concerns | Moderate confidence  **Explanation:** Moderate concerns regarding adequacy because of thin data from few studies. No or very minor concerns regarding methodological limitations, coherence, adequacy, and relevance. | Abukari & Acheampong 2021; Cohen et al. 2017; Daniel et al. 2019; |
