# Supplementary material for: Factors that influence the provision of enteral feeding for critically ill children: a qualitative evidence synthesis
Source: BMC Nutr. 2025 May 19;11:98. doi: 10.1186/s40795-025-01077-3 (PMC12087210; doi:10.1186/s40795-025-01077-3)
Supplement: Supplementary file 7 — Additional file 7: Implications for practice. [file 40795_2025_1077_MOESM7_ESM.pdf]

Enteral feeding for critically ill children: Implications for practice

The following questions are intended for policymakers, hospital staff, managers, and others who are involved in the planning and implementation of enteral feeding for critically ill children. The questions are based on our review findings and address issues that the research suggests are important to parents, children and healthcare workers. These prompts are not intended to be recommendations but are phrased as questions to help implementers consider the implications of the review findings within their context.

1. How can you ensure that healthcare workers have access to nutritional training and support?

- Do hospital staff have **regular training** in feeding critically ill children? Does the timing of training take staff turnover into consideration? Does the training cover how to give information and support to parents, how to insert an enteral feeding tube and how to give feeds?
- Do staff have easy and timely **access to dietitians**, nutritionists or other appropriate personnel to consult as needed, including on children's nutritional requirements and on appropriate feeds?
- Do staff have **protocols** for enteral feeding of critically ill children? Do they have tools to calculate nutritional needs? And have they received training in the use of these protocols and tools?

2. How can you ensure that healthcare facilities have reliable access to feeds and equipment?

- In some settings, specialised resources for enteral feeding are available only in departments that treat malnourished children. Do all hospital wards where enteral feeding is used have the necessary **equipment** to provide and manage the delivery of enteral feeding to children? Do all hospitals have the **funding** they need to cover the costs of enteral feeding for all wards that implement this, for instance, for commercial or specialised feeds and equipment?

3. How can you provide parents and children with adequate information and support at different stages of the enteral feeding process?

- Parents and children may find the tube insertion process uncomfortable or distressing, but enteral feeding sometimes takes place in situations where there is little time to give parents and children information and support. Consider how best to **prepare children and parents** for the insertion process, particularly in busy hospital wards. For instance, can staff explain what will happen and whether it will cause discomfort? Can parents be given the option of not being present when the tube is inserted if they feel it will be distressing?
- Hospital staff do not always have the knowledge and time to provide support or information and answer questions. In addition, parents may not remember information that is given when they are under stress. Can **information be provided in a format that parents (and hospital staff) can access** at any time point, for instance information leaflets, webpages or wall posters? Have you considered how to provide information to people who don't speak the majority language or don't read or write, for instance pictorial information?

- Parents may find it difficult to see their child being fed through a tube and may interpret it as an indication that the child is terminally ill or sicker than they actually are. How can staff provide parents with the **reassurance and support** they need?
- In addition to the support staff can provide, **parents can be sources of support for each other**. Can dedicated spaces be provided in the hospital where parents can meet each other? Can parents get help to establish or access peer support groups?
- Parents and older children are also likely to have questions after enteral feeding has started. Do parents have easy **access to information** that can answer questions including:
    - Why can't my child eat?
    - Is the tube causing pain and discomfort?
    - How will my child's skin react to the adhesive tape that may be used to fit the tube in place?
    - Will there be any side effects?
    - Will enteral feeding impact on my child's growth?
    - How long will the tube stay in?
    - What can I do if my child experiences vomiting or nausea?

4. How can you ensure that parents are involved in the decision-making process?

- Have staff ensured that parents have given **consent**?
- Do staff have the skills and time they need to **involve parents in the decisions**? Have parents received enough information to participate meaningfully in decisions about, for example, type of enteral feeding, type of feed or how often the feed is given?
- Could **shared decision-making aids** be made available?

5. How can you support mothers who were breastfeeding when enteral feeding started?

- How can hospital staff help mothers develop or maintain **bonding with their child** when breastfeeding is not possible?
- How can staff help breastfeeding mothers **maintain their breast milk supply**? For instance, can they offer advice and support about how to express breastmilk or use a pump?
- Do mothers have easy **access to a pump**? In hospital wards where the milk is not used immediately, are there appropriate storage facilities for their breast milk?
- Can **designated spaces** be provided for mothers to have privacy when expressing milk? Mothers may be concerned if the breastmilk that they expressed was not used, or if their child is given other feeds than breastmilk. Do hospital staff take care to use the breastmilk or to **explain** why the milk was not used? If additional feeds are provided, do hospital staff **explain** to mothers why the breastmilk is not sufficient?
