# Supplementary material for: Factors that influence the provision of enteral feeding for critically ill children: a qualitative evidence synthesis
Source: BMC Nutr. 2025 May 19;11:98. doi: 10.1186/s40795-025-01077-3 (PMC12087210; doi:10.1186/s40795-025-01077-3)
Supplement: Supplementary file 8 — Additional file 8: References to excluded studies. [file 40795_2025_1077_MOESM8_ESM.docx]

***Additional file 8. References to excluded studies***

1. Abeasi DA, Emelife B. What mothers go through when the unexpected happens: A look at challenges of mothers with preterm babies during hospitalization in a tertiary institution in Nigeria. Journal of Nursing and Midwifery Sciences. 2020;7(1):22-9.

2. Abuidhail J, Al-Motlaq M, Mrayan L, Salameh T. The lived experience of Jordanian parents in a neonatal intensive care unit: A phenomenological study. Journal of Nursing Research. 2017;25(2):156-62.

3. Beck CT, Woynar J. Posttraumatic stress in mothers while their preterm infants are in the newborn intensive care unit: A mixed research synthesis. Advances in Nursing Science. 2017;40(4):337-55.

4. Bicakli DH, Yildirim Sari H, Yilmaz M, Cetingul N, Kantar M. Paediatric oncology patients' and their mothers' enteral nutrition experiences: A qualitative study. Pediatric Blood and Cancer. 2016;63(Supplement 3):S220.

5. Brotherson MJ, Oakland MJ, Secrist-Mertz C, Litchfield R, Larson K. Quality of Life Issues for Families who Make the Decision to Use a Feeding Tube for Their Child with Disabilities. Journal of the Association for Persons with Severe Handicaps. 1995;20(3):202-12.

6. Brotherton A, Abbott J. Clinical decision making and the provision of information in PEG feeding: An exploration of patients and their carers'perceptions. Journal of Human Nutrition and Dietetics. 2009;22(4):302-9.

7. Brotherton A, Abbott J, Hurley M, Aggett PJ. Home enteral tube feeding in children following percutaneous endoscopic gastrostomy: Perceptions of parents, paediatric dietitians and paediatric nurses. Journal of Human Nutrition and Dietetics. 2007;20(5):431-9.

8. Brotherton AM, Abbott J, Aggett PJ. The impact of percutaneous endoscopic gastrostomy feeding in children; the parental perspective. Child Care Health Dev. 2007;33(5):539-46.

9. Bucher HU, Klein SD, Hendriks MJ, Baumann-Hölzle R, Berger TM, Streuli JC, et al. Decision-making at the limit of viability: Differing perceptions and opinions between neonatal physicians and nurses. BMC Pediatrics. 2018;18(1).

10. Bunyani A, Mtimuni B, Kalimbira A, Kamalo P. Experiences of health professionals with nutritional support of critically ill patients in tertiary hospitals in Malawi. Malawi Medical Journal. 2015;27(1):1-4.

11. Chaplen C. Parents' views of caring for children with gastrostomies. British journal of nursing (Mark Allen Publishing). 1997;6(1):34-8.

12. Cipolla J, Reeves-Latour J, Ramsay M, Li P. Mothers' experiences and perceptions of their child's weaning process from tube feeding within a multidisciplinary Paediatric Feeding Program. Paediatrics and Child Health (Canada). 2022;27(6):353-8.

13. Craig GM. Psychosocial aspects of feeding children with neurodisability. European Journal of Clinical Nutrition. 2013;67:S17-S20.

14. Craig GM, Scambler G, Spitz L. Why parents of children with neurodevelopmental disabilities requiring gastrostomy feeding need more support. Dev Med Child Neurol. 2003;45(3):183-8.

15. Dadich A, Hockey K, Kaplun C, Fleming C, Hopwood N, Moraby K, et al. Clinician and carer moral concerns when caring for children who tube-feed. Journal of Child Health Care. 2023;27(2):182-96.

16. Depianti JRB, Cabral IE. Hospitalized children with complex special healthcare needs: multiple case studies. ACTA Paulista de Enfermagem. 2023;36.

17. Edwards S, Davis AM, Bruce A, Mousa H, Lyman B, Cocjin J, et al. Caring for tube-fed children: A review of management, tube weaning, and emotional considerations. Journal of Parenteral and Enteral Nutrition. 2016;40(5):616-22.

18. Fleming CAK, Cohen J, Murphy A, Wakefield CE, Cohn RJ, Naumann FL. Parent feeding interactions and practices during childhood cancer treatment. A qualitative investigation. Appetite. 2015;89:219-25.

19. Guerriere DN. Measuring decisional conflict in substitute decision makers: Mothers' decisions about initiating gastrostomy tube feeding in children. Measuring Decisional Conflict in Substitute Decision Makers: Mothers' Decisions About Initiating Gastrostomy Tube Feeding in Children. 1998.

20. Guerriere DN, McKeever P, Llewellyn-Thomas H, Berall G. Mothers' decisions about gastrostomy tube insertion in children: Factors contributing to uncertainty. Dev Med Child Neurol. 2003;45(7):470-6.

21. Gunes NEO, Cetinkaya S. Assessment the knowledge, care, and experiences of neonatal nurses about enteral nutrition. Medicine. 2023;102(21):e31081.

22. Halse C, Boughtwood D, Clarke S, Honey A, Kohn M, Madden S. Illuminating multiple perspectives: meanings of nasogastric feeding in anorexia nervosa. European Eating Disorders Review. 2005;13(4):264-72.

23. Herrington P, Assey J, Rouse L, Baker R. Gastrostomy and children: A review of the literature in learning disabilities. Children and Society. 2001;15(5):375-86.

24. Hopwood N, Elliot C, Moraby K, Dadich A. Parenting children who are enterally fed: How families go from surviving to thriving. Child: Care, Health and Development. 2020;46(6):741-8.

25. Hopwood N, Pointon K, Dadich A, Moraby K, Elliot C. Forward anchoring in transformative agency: How parents of children with complex feeding difficulties transcend the status quo. Learning, Culture and Social Interaction. 2022;33.

26. Huhmann MB, August DA. Review of American Society for Parenteral and Enteral Nutrition (A.S.P.E.N.) clinical guidelines for nutrition support in cancer patients: Nutrition screening and assessment. Nutrition in Clinical Practice. 2008;23(2):182-8.

27. Keller HH, Vesnaver E, Davidson B, Allard J, Laporte M, Bernier P, et al. Providing quality nutrition care in acute care hospitals: Perspectives of nutrition care personnel. Journal of Human Nutrition and Dietetics. 2014;27(2):192-202.

28. Kodua M, Mackenzie J-M, Smyth N. Nursing assistants’ experiences of administering manual restraint for compulsory nasogastric feeding of young persons with anorexia nervosa. International Journal of Mental Health Nursing. 2020;29(6):1181-91.

29. Leong AY, Cartwright KR, Guerra GG, Joffe AR, Mazurak VC, Larsen BMK. A Canadian survey of perceived barriers to initiation and continuation of enteral feeding in PICUs. Pediatric Critical Care Medicine. 2014;15(2):e49-e55.

30. Lindberg N, Berglund AL. Mothers' experiences of feeding babies born with cleft lip and palate. Scandinavian Journal of Caring Sciences. 2014;28(1):66-73.

31. Lively EJ, McAllister S, Doeltgen SH. Characterizing International Approaches to Weaning Children From Tube Feeding: A Scoping Review. Journal of Parenteral and Enteral Nutrition. 2021;45(2):239-50.

32. Mahant S, Cohen E, Nelson KE, Rosenbaum P. Decision-making around gastrostomy tube feeding in children with neurologic impairment: Engaging effectively with families. Paediatrics and Child Health (Canada). 2018;23(3):209-13.

33. Mahant S, Jovcevska V, Cohen E. Decision-making around gastrostomy-feeding in children with neurologic disabilities. Pediatrics. 2011;127(6):e1471-e81.

34. Michaelis CA, Warzak WJ, Stanek K, Van Riper C. Parental and professional perceptions of problems associated with long- term pediatric home tube feeding. Journal of the American Dietetic Association. 1992;92(10):1235-8.

35. Morris G, Anthony A, Adrian T. Barriers to exclusive enteral nutrition for induction of remission of childhood crohn's disease: Qualitative analysis of a randomised controlled. Inflammatory Bowel Diseases. 2014;20(SUPPL. 1):S82.

36. Morrow AM, Quine S, Loughlin EV, Craig JC. Different priorities: a comparison of parents' and health professionals' perceptions of quality of life in quadriplegic cerebral palsy. Arch Dis Child. 2008;93(2):119-25.

37. Murphy N, Ravikumara M, Butterworth M, Glasson E, Rennison C, Prevett B, et al. A Co-Designed Online Education Resource on Gastrostomy Feeding for Parents and Caregivers to Support Clinical Care. Journal of pediatric gastroenterology and nutrition. 2023;77(5):672-8.

38. Namnabati M, Hemati Z, Taleghani F, Sadeghnia A. Home-based care needs of preterm infants discharged early from the neonatal intensive care unit: A descriptive qualitative study. Iran J Neonatol. 2017;8(4):74-82.

39. Neiderman M, Farley A, Richardson J, Lask B. Nasogastric feeding in children and adolescents with eating disorders: Toward good practice. International Journal of Eating Disorders. 2001;29(4):441-8.

40. Nelson KE, Lacombe-Duncan A, Cohen E, Nicholas DB, Rosella LC, Guttmann A, et al. Family experiences with feeding tubes in neurologic impairment: A systematic review. Pediatrics. 2015;136(1):e140-e51.

41. Pinch WJ. Five families share their views of ethical decisionmaking in the NICU. Caring : National Association for Home Care magazine. 1990;9(12):12-8.

42. Poh PF, Carey MC, Manning JC, Lee JH, Latour JM. Parental emotional, social and transitional health in the first 6 months after childhood critical illness: A longitudinal qualitative study. Journal of Advanced Nursing. 2024.

43. Radford MJ, Thorne S, Bassingthwaighte C. Long-term gastrostomy in children: Insights from expert nurses. Comprehensive Child and Adolescent Nursing. 1997;20(1):35-50.

44. Ridley EJ, Chapple LAS, Chapman MJ. Nutrition intake in the post-ICU hospitalization period. Current Opinion in Clinical Nutrition and Metabolic Care. 2020;23(2):111-5.

45. Rouse L, Herrington P, Assey J, Baker R, Golden S. Feeding problems, gastrostomy and families: A qualitative pilot study. British Journal of Learning Disabilities. 2002;30:122-8.

46. Sleigh G. Mothers' voice: a qualitative study on feeding children with cerebral palsy. Child Care Health Dev. 2005;31(4):373-83.

47. Soscia J, Adams S, Cohen E, Moore C, Friedman JN, Gallagher K, et al. The parental experience and perceptions of blenderized tube feeding for children with medical complexity. Paediatr Child Health. 2021;26(8):462-9.

48. Spalding K, McKeever P. Mothers' experiences caring for children with disabilities who require a gastrostomy tube. J Pediatr Nurs. 1998;13(4):234-43.

49. Sullivan PB. Gastrostomy and the disabled child. Dev Med Child Neurol. 1992;34(6):552-5.

50. Swanson V, Nicol H, McInnes R, Cheyne H, MacTier H, Callander E. Developing maternal self-efficacy for feeding preterm babies in the neonatal unit. Qualitative Health Research. 2012;22(10):1372-82.

51. Syrmis M, Frederiksen N, Reilly C. Characterisation of information Hospitals Provide Parents on Tube Feeding, Including Tube Weaning. Journal of Pediatric Nursing. 2019;44:e91-e7.

52. Thomas S, Morrison A, Morton G, Roberts P, Clark V, Imrie J. The burden of disease in metachromatic leukodystrophy: results of a caregiver survey in the UK and Republic of Ireland. Orphanet journal of rare diseases. 2024;19(1):87.

53. Townsley R, Robinson C. More than just a health issue: A review of current issues in the care of enterally-fed children living in the community. Health and Social Care in the Community. 1999;7(3):216-24.

54. Wigert H, Johansson R, Berg M, Hellström AL. Mothers' experiences of having their newborn child in a neonatal intensive care unit. Scandinavian Journal of Caring Sciences. 2006;20(1):35-41.

55. Wilken M. The Impact of Child Tube Feeding on Maternal Emotional State and Identity: A Qualitative Meta-Analysis. Journal of Pediatric Nursing. 2012;27(3):248-55.
